# (Re-)Directing Oligomerization of a Single Building Block into Two Specific Dynamic Covalent Foldamers through pH

**DOI:** 10.1021/jacs.2c09325

**Published:** 2023-01-27

**Authors:** Yulong Jin, Pradeep K. Mandal, Juntian Wu, Niklas Böcher, Ivan Huc, Sijbren Otto

**Affiliations:** † Beijing National Laboratory for Molecular Sciences, CAS Key Laboratory of Analytical Chemistry for Living Biosystems, Institute of Chemistry, Chinese Academy of Sciences, 100190 Beijing, China; ‡ Centre for Systems Chemistry, Stratingh Institute, Nijenborgh 4, 9747 AG Groningen, The Netherlands; § Department of Pharmacy and Center for Integrated Protein Science, Ludwig-Maximilians Universität, 81377 Munich, Germany

## Abstract

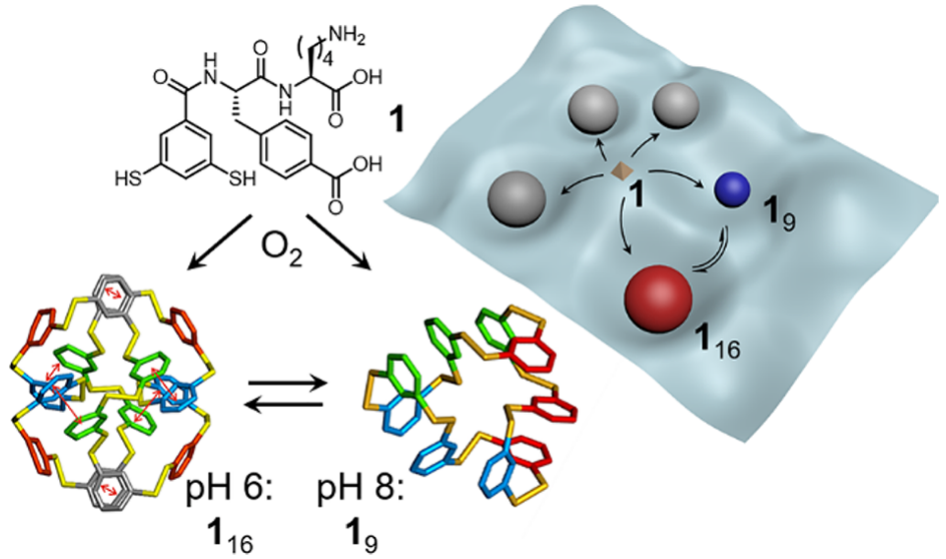

Dynamic foldamers are synthetic folded molecules which can change their conformation in response to an external stimulus and are currently at the forefront of foldamer chemistry. However, constitutionally dynamic foldamers, which can change not only their conformation but also their molecular constitution in response to their environment, are without precedent. We now report a size- and shape-switching small dynamic covalent foldamer network which responds to changes in pH. Specifically, acidic conditions direct the oligomerization of a dipeptide-based building block into a 16-subunit macrocycle with well-defined conformation and with high selectivity. At higher pH the same building block yields another cyclic foldamer with a smaller ring size (9mer). The two foldamers readily and repeatedly interconvert upon adjustment of the pH of the solution. We have previously shown that addition of a template can direct oligomerization of the same building block to yet other rings sizes (including a 12mer and a 13mer, accompanied by a minor amount of 14mer). This brings the total number of discrete foldamers that can be accessed from a single building block to five. For a single building block system to exhibit such highly diverse structure space is unique and sets this system of foldamers apart from proteins. Furthermore, the emergence of constitutional dynamicity opens up new avenues to foldamers with adaptive behavior.

## Introduction

Evolution has produced folded biomacromolecules of impressive structural complexity that now fulfill central roles in biochemistry. In many instances the proper functioning of these molecules (in, for example, catalysis or signal transduction) requires them to change their conformation in response to cues such as binding to signaling molecules or other biomacromolecules or changes in environment.^1−4^ Conformational dynamics can also be engineered into existing proteins through synthetic biology approaches, leading to protein switches which can be modulated by environmental cues such as pH.^5−8^ Inspired by such structures and their behavior, chemists have designed and synthesized folded molecules from scratch, based on natural building blocks as well as synthetic analogs, mostly targeting helical folds or β-sheet mimics.^9−12^ More recently, efforts have been directed at developing foldamers that can adopt different conformations, depending on environmental cues such as guest binding or photoirradiation.^18−30^ In many cases these systems are switched between helical conformations of different handedness, or between folded and unfolded states. Switching between structurally more different conformations has until now remained rare.^31−34^ Previous studies also showed that macrocycles, catenanes, and knots synthesized by dynamic covalent chemistry are responsive to chemical stimuli or templates.^13−17^

**Scheme 1:**
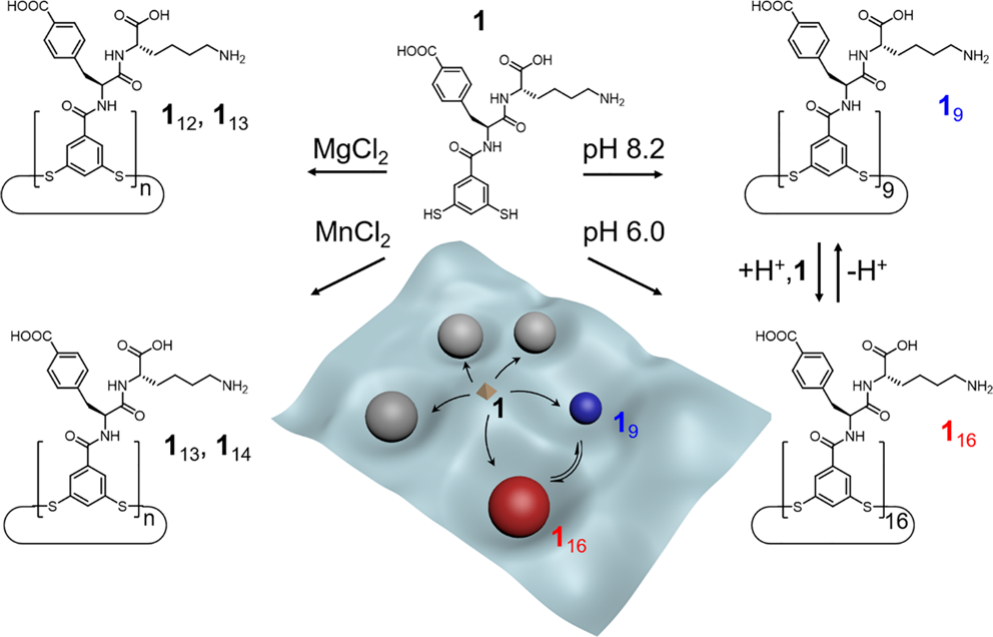
Emergence of, and pH-Induced Interconversion between Constitutionally Different Foldamers Derived from the Same Monomer As previously reported,^39^ adding MgCl_2_ or MnCl_2_ as templates directs the oligomerization of building block **1** to produce different foldamers (mixtures of **1**_12_ and **1**_13_ or of **1**_13_ and **1**_14_). We now show that building block **1** can form a 16mer at pH 6.0 and can reversibly switch between 16mer and 9mer (which dominates at pH 8.2) as a function of the pH.

We recently exploited a dynamic combinatorial approach^35,36^ to foldamer formation,^37,38^ using reversible covalent chemistry to oligomerize relatively simple building blocks.^39,40^ Folding was found to selectively stabilize specific oligomers, causing the equilibrium to shift in favor of folded molecules. In principle, this approach would allow the discovery of foldamers that not only change their structure but also change their molecular constitution in response to a change in their environment. We already demonstrated previously that guests can direct foldamer formation in dynamic combinatorial libraries prepared from building block **1** (Scheme 1).^39^ Under the conditions typically used in aqueous reversible disulfide chemistry (pH around 8) this building block forms predominantly a cyclic nonamer driven by its folding. Upon exposure to suitable guest molecules the equilibrium shifts to produce mainly 12- or 13-membered macrocycles. It was already remarkable that different foldamers could be accessed from a single building block. We now report that the same building block, at a different pH, gives rise to yet another foldamer, composed of 16 subunits of **1**. Upon changing the pH, and provided a sufficient amount of thiolate is present, the system can be switched cleanly and repeatedly between the 9mer and 16mer structures. The switchover takes place over a remarkably narrow pH range. The crystal structures of **1**_9_ and **1**_16_ reveal completely different folds and highlight how folding affects the microenvironment of some ionizable groups, which determines how the stability of these two foldamers may be influenced by the pH.

## Results and Discussion

**Figure 1:**
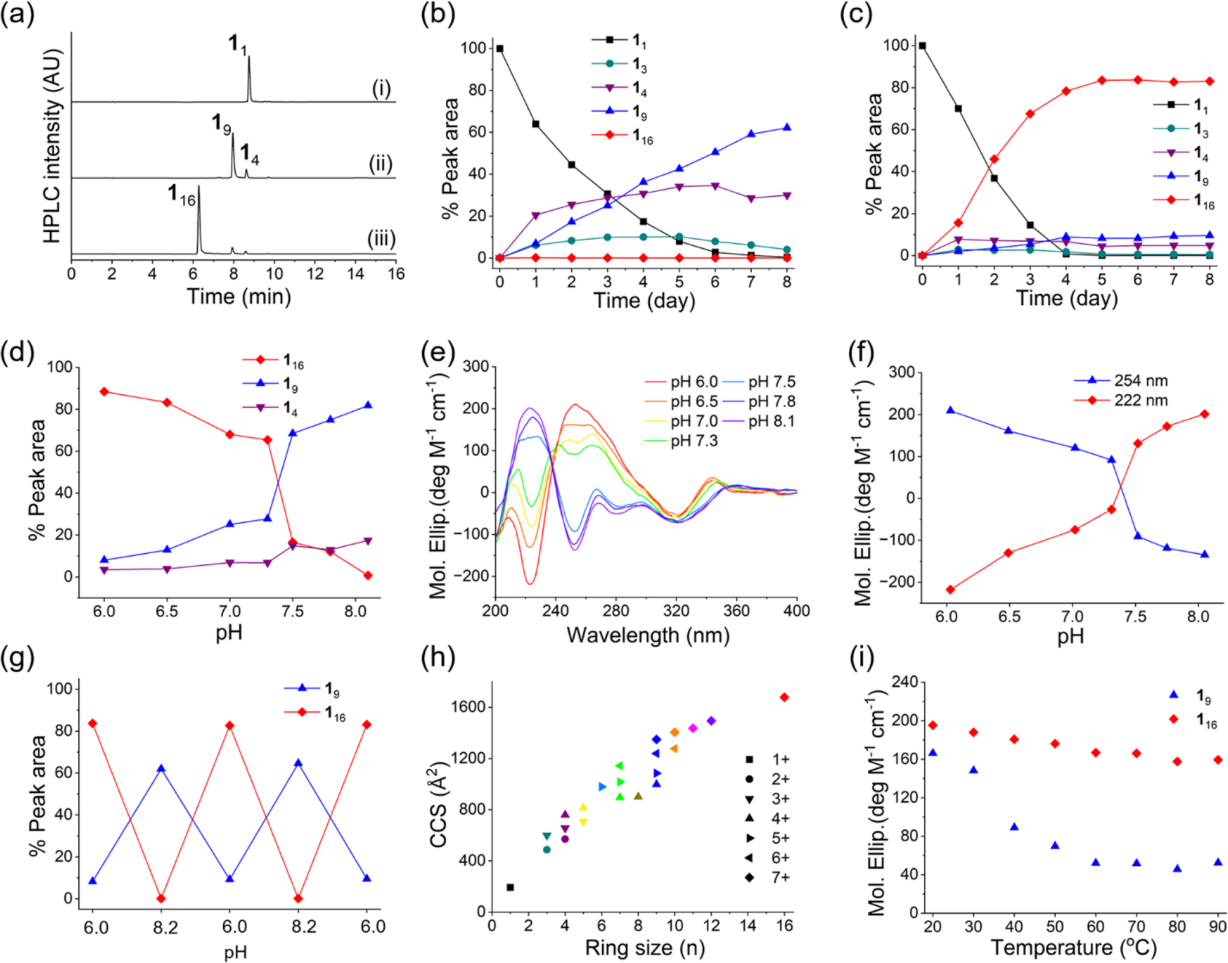
Characterization of the pH responsiveness of the constitutional dynamic foldamers. (a) UHPLC chromatograms (absorbance at 254 nm) of a small dynamic combinatorial library made from building block **1** (1.0 mM) in 25 mM phosphate buffer (i) at pH 8.2 at *t* = 0, (ii) on day 6, and (iii) at pH 6.0 on day 6. Kinetic profiles for the above library at (b) pH 8.2 and (c) pH 6.0. (d) Relative peak areas of **1**_4_, **1**_9_, and **1**_16_ as a function of pH; UHPLC chromatograms are shown in Figure S8. (e) CD spectra of the libraries at different pHs. (f) Change of the CD signals characteristic of **1**_9_ (222 nm) and **1**_16_ (254 nm) with pH. (g) Interconversion between the **1**_16_ and **1**_9_ foldamers in response to a change in pH. Note that addition of **1** is required for converting **1**_9_ to **1**_16._ (h) Collision cross sections of the different macrocycles determined by UPLC-ion mobility-mass spectrometry. All macrocycles that are detected by mass spectrometry have been included in the graph. Symbol shapes indicate the different charge states, with the same ring size in the same color. (i) Temperature-dependent CD data showing the changes of the absolute value of the molar ellipticity at 254 nm of isolated **1**_9_ and **1**_16_ upon elevating the temperature from 20 to 90 °C. Samples of **1**_9_ and **1**_16_ were prepared in 25 mM phosphate buffer at pH 8.2 and pH 6.0, respectively. Lines between datapoints are drawn to guide the eye.

When searching for systems where the environment would affect folding, we homed in on building block **1**, as we previously observed that this building block gives rise to a folded nine-membered disulfide-linked macrocycle (Figure 1b) that unfolds relatively readily, indicating that it occupies a relatively shallow energy minimum (Scheme 1).^39^ Specifically, significant changes in its NMR spectrum were observed upon heating to 65 °C, which were reverted upon cooling. Thus, compared to the other foldamers we discovered previously that did not undergo such transition, it might be easier to affect folding by changing experimental conditions. Altering the pH would be a suitable environmental perturbation, given the number of ionizable groups of **1** that will be brought into close proximity upon oligomerization and folding.

### pH-Dependent Emergence of Two Different Foldamers

Indeed, when allowing oligomerization of building block **1** at pH 6.0, a new 16-membered macrocycle emerged from the small dynamic combinatorial library, eventually occupying more than 85% of the total peak area (Figure 1a and c). This amounts to a 1400-fold amplification of this compound when compared to its amount detected at pH 8.2. We also analyzed the library composition at intermediate pHs, which revealed that the transition from forming one folded structure to forming another was remarkably sharp, within about 0.2 pH unit, reminiscent of a phase transition, suggesting a cooperative process (Figure 1d and Figure S8). Incidentally, the transition occurs near physiological pH.

We further characterized the samples by circular dichroism (CD), showing that **1**_9_ and **1**_16_ adopt distinct conformations (Figure 1e). Analysis of the samples prepared at increasing pHs showed that the signal at 222 nm increased and the signal at 254 nm decreased, in agreement with **1**_16_ dominating at lower pH and **1**_9_ at higher pH (Figure 1f).

### Foldamers **1**_9_ and **1**_16_ Can Be Interconverted

Having established that different foldamers emerge at different pH, we probed whether, once formed, these compounds could be interconverted in response to changing the pH. We first prepared **1**_16_ at pH 6.0 (in 25 mM phosphate buffer), where it accounted for over 80% of the library material after 5 days, as shown in Figure 1g. We then changed the pH to 8.2 (also in 25 mM phosphate buffer). In order to avoid an increase in ionic strength which would result from the addition of base, we exchanged the buffer using a 3K centrifugal filter, which retains the foldamers while allowing the buffer to pass through. After a washing step, the residue was then taken up in the pH 8.2 buffer, where **1**_16_ converted into **1**_9_ within 1 day, with **1**_9_ accounting for 63% of the total peak area. Foldamer **1**_9_ could be reverted to **1**_16_ by reducing the pH back to 6.0 using a similar protocol, while also adding 5 mol% of monomer **1** to ensure efficient disulfide exchange. Comparable results were obtained upon adding 5 mol% tris(2-carboxyethyl)phosphine (TCEP) into a mixture dominated by **1**_9_ at pH 6.0. In the absence of **1** no interconversion was observed on the same timescale, suggesting that **1**_9_ is kinetically trapped under these conditions.

The fact that the interconversion of **1**_9_ to **1**_16_ requires addition of thiol, while the reverse process does not, bestows the system with a “memory” of its history. If no thiol is added and the system has been at pH 8.2, it will remain in the **1**_9_ state when the pH is reduced to 6.0, even though at this pH **1**_16_ is its most stable state. We attribute the requirement for addition of thiol when switching between foldamers at pH 6.0 to the reduced availability of thiolate anion (which mediates disulfide exchange) under these more acidic conditions.

The dynamic interconversion between the two foldamers could be repeated without loss in efficiency, reaching essentially identical compositions after each cycle (Figure 1g). We also investigated the dynamic covalent interconversion at a higher salt concentration (50 mM phosphate buffer, pH 6.0 and 8.2). Under these conditions **1**_16_ was smoothly converted to **1**_9_, but this transition could not be reversed, suggesting that the formation of **1**_16_ is hampered by a high ionic strength. We speculate that intramolecular salt bridge interactions play a role in the formation of the foldamers and that they are more prevalent in **1**_16_ than in **1**_9_.

### Foldamer Characterization by Mass Spectroscopy, Circular Dichroism, and NMR

Ion mobility-mass spectrometry (IM-MS) is a potentially useful tool for obtaining structural information on macromolecules in the gas phase because of its ability to provide the rotationally averaged collision cross-section (CCS) of the molecules, which is related to the extent that they are compacted by folding.^41,42^ We subjected a dynamic combinatorial library made from **1** containing a range of different macrocycles to ultra-performance liquid chromatography (UPLC)-IM-MS analysis and determined the CCS of these compounds at different charge states (Figure 1h). Similar to previous observations for a related foldamer system,^39^ the CCS of a particular ring correlated with its charge state, with larger CCS at higher charge states, in agreement with molecules becoming less compact as intramolecular charge repulsion increases in the gas phase. Given the relatively large effect of the charge state on the CCS and the relatively small range of macrocycles that can be observed with the same charge state, drawing further conclusions from the IM-MS data is difficult.

In order to obtain additional information on the structures of **1**_9_ and **1**_16_, these compounds were purified by semi-preparative high-performance liquid chromatography (HPLC). Ultra-high-performance liquid chromatography (UHPLC) analysis indicates a purity higher than 95%, as shown in Figures S9–S11. These samples were then characterized by CD spectroscopy. As shown in Figure S12, the CD spectrum of **1**_16_ has an intense positive band at 254 nm, which can be attributed to the absorption by the aromatic dithiol and carboxylphenylalanine side chain. The negative band at 222 nm is attributed to the absorption by the amide bonds. The 9mer shows a negative band at 254 nm and a positive band at 222 nm. The reversal in sign of the CD bands when comparing **1**_9_ to **1**_16_ suggests that the two macrocycles adopt opposite screw senses in their three-dimensional conformation. Importantly, the molar ellipticities (calculated in units of **1**) of **1**_9_ and **1**_16_ are dramatically enhanced compared to those of monomer and tetramer, which suggests that the aromatic rings in **1**_9_ and **1**_16_ reside in well-defined chiral environments in both macrocycles. Temperature-dependent CD experiments showed that the CD signals (254 nm) of **1**_16_ decreased by only 18% upon heating from 20 to 90 °C, indicating that the 16mer has a high thermostability (Figure 1i, Figure S13). In contrast, the 9mer starts to unfold at a lower temperature (30 °C), and the absolute intensity of the CD signal at 254 nm decreases by 68.5% across the same temperature range, indicative of a less stably folded structure (Figure 1i, Figure S14). After a heat–cool cycle, the ellipticity of **1**_16_ was fully recovered (Figure S13). For **1**_9_, the signal at 254 nm was also recovered but to a reduced extent (82%) (Figure S14), providing a further indication of the higher robustness of **1**_16_. Subsequent UHPLC analysis showed that both compounds remained chemically unchanged after the heat–cool cycles.

**Figure 2:**
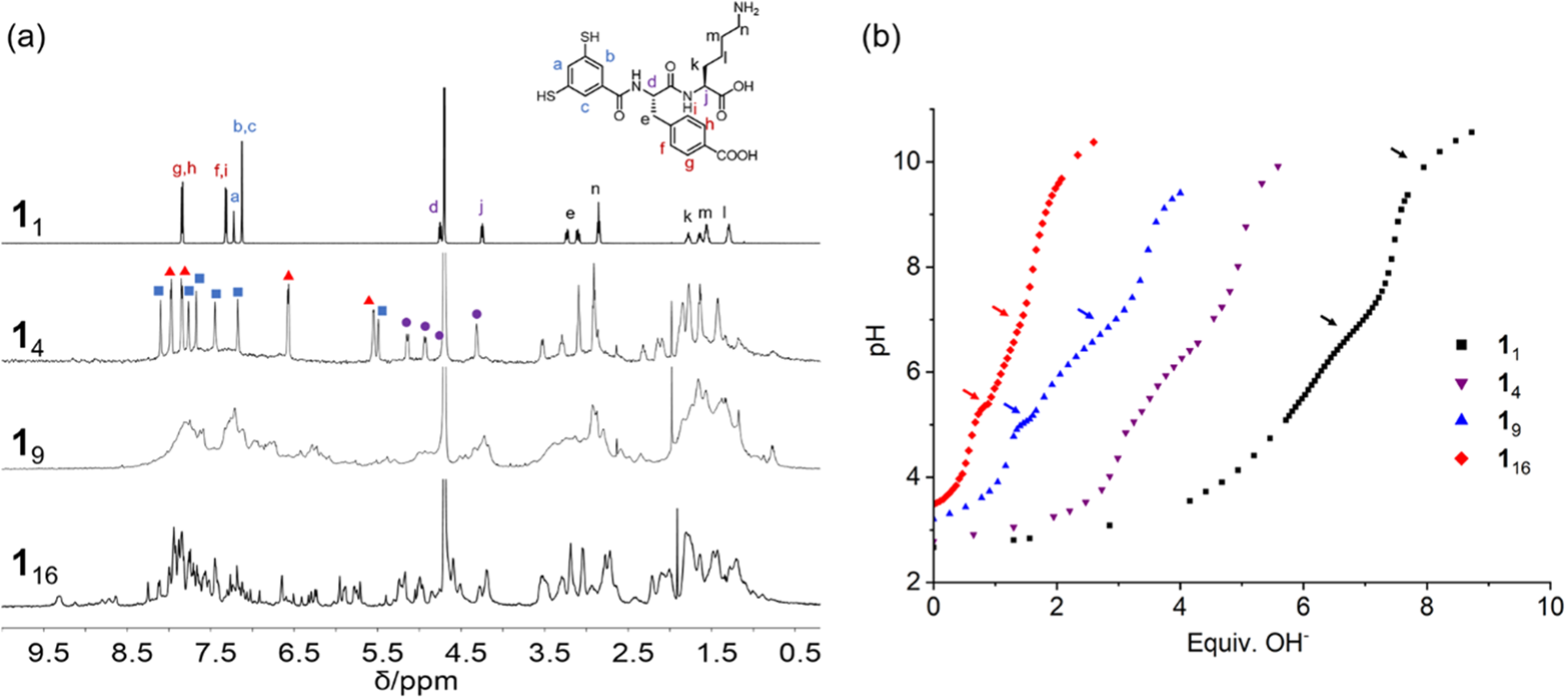
^1^H NMR spectra and pH titrations. (a) ^1^H NMR spectra of dithiol building block **1** and disulfide macrocycles **1**_4_, **1**_9_, and **1**_16_ in D_2_O at room temperature (600 MHz). (b) pH titration of building block **1** and macrocycles **1**_4_, **1**_9_, and **1**_16_ in water. The *x*-axis shows the number of equivalents of OH^–^ added per monomer. Arrows indicate the buffering regions. Note that the titrations of the different macrocycles started from samples made at different pH values (2.67 for **1**, 2.78 for **1**_4_, 3.21 for **1**_9_, and 3.48 for **1**_16_) so that the curves do not overlap.

Solution-phase ^1^H NMR spectra (D_2_O, 298 K) of the two foldamers, as well as of tetramer **1**_4_, are shown in Figure 2a. The tetramer shows sharp signals which appear in two separate sets, indicative of a *C*_2_ symmetry for this molecule in solution. The protons of the phenyl rings are spread over a wide range of chemical shifts (from 5.5 to 8.2 ppm) suggesting that some of these protons are situated near the face of other aromatic rings. Apparently, this small macrocycle also adopts a specific conformation which is stable on the NMR timescale. The 9mer revealed broad signals indicating a less ordered conformation. In contrast, the 16mer showed remarkably sharp peaks suggesting a well-defined and highly ordered structure in solution. This difference suggests that in solution **1**_9_ has more conformational freedom than **1**_16_. Temperature-dependent CD data (Figure 1i) support this hypothesis as it indicates that **1**_9_ unfolds more readily upon heating than **1**_16_. The upfield chemical shift of some phenyl protons detected for **1**_9_ and **1**_16_ indicates that they are close to an aromatic ring. The complexity of the spectrum, combined with the fact that **1**_16_ consists of 16 identical monomer units, makes further structural elucidation by NMR highly challenging.

### pH Titrations Suggest Folding Perturbs p*K*_a_ Values

Given the large effects of pH and ionic strength on foldamer formation and interconversion, we decided to perform pH titrations on building block **1** and macrocycles **1**_4_, **1**_9_ and **1**_16_ in order to determine the p*K*_a_ values of the various ionizable groups in these structures (Figure 2b). First, the stability of the HPLC-purified foldamers (**1**_16_ and **1**_9_, purity higher than 95%) was monitored in an aqueous solution at pH 3.0 and pH 8.2. In the absence of thiol or reducing agent they remained intact for at least 24 h. Titrations started from acidic solutions. The buffering capacity of monomer **1** at around pH 7.0 and pH 10.5 is attributed to proton transfer from and to aromatic thiols (p*K*_a_ ≈ 7) and lysine amine groups (p*K*_a_ ≈ 10.5), respectively. For **1**_9_ and **1**_16_ we expected that the deprotonation of the first carboxylic acid residue should occur more readily (i.e., at a lower p*K*_a_ than the range of 4.2–4.7 typical of carboxylic acids) as it alleviates the electrostatic repulsion due to the protonated lysine residues. The buffering region observed below pH 4.0 is likely to correspond to this ionization event. Buffering capacities at pH 5.1 and pH 5.4 were detected for **1**_9_ and **1**_16_, respectively, suggesting an elevated p*K*_a_ of the carboxylic acid groups. Such elevated p*K*_a_ values suggest that the carboxylic acid groups are situated closely in space, suppressing their ionization. Less pronounced plateaus were also detected in the range between pH 7.0 and pH 7.5 for **1**_9_ and **1**_16_, indicating the existence of a third subset of carboxylic acid groups with much-upshifted p*K*_a_ values. This p*K*_a_ range coincides with the pH range (pH 7.3–7.5) where the switchover between the two foldamers occurs, suggesting that these groups are implicated in causing the difference in stability between the two foldamers at different pHs. In contrast, for the monomer and tetramer no buffering capacity was detected at this pH range, indicating that the p*K*_a_ values of the carboxylic acid groups are less perturbed in these compounds. Note that analysis by HPLC confirmed that all compounds remained unchanged after the pH titration experiments, indicating that no constitutional changes or decomposition occurred during the titrations.

### Structural Elucidation by X-ray Crystallography and Rationalization of the pH-Driven Constitutional Switch

**Figure 3:**
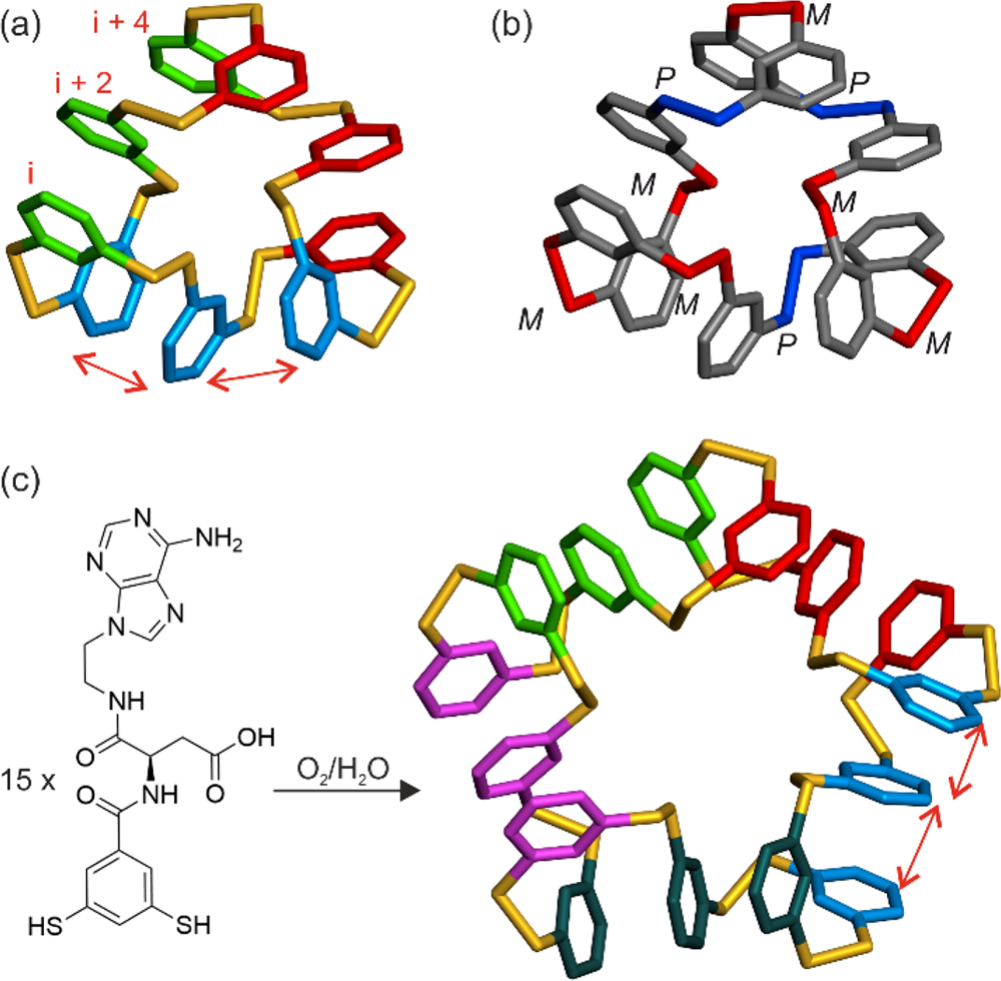
Crystal structure of **1**_9_. (a) Color-coded tube representation of the hydrophobic dimercaptobenzene core showing three sets of rings at the *i*, *i*+2, and *i*+4 positions stacked face-to-face. Disulfide bonds are shown in yellow. Hydrogen atoms have been omitted for clarity. (b) Same view with the benzene rings shown in gray and the disulfide bonds in red or blue, depending on their *M* or *P* chirality, respectively, clearly showing the lack of symmetry of the structure. The unit cell also contains the enantiomer d-**1**_9_. (c) An analogous pentagonal structure formed from the assembly of 15 units of a monomer bearing an amino acid and a nucleobase.^40^ Note that the chirality of both the monomer formula and the pentadecamer structure has been inverted with respect to the original publication to facilitate the comparison with the structure of **1**_9_.

Crystals suitable for solving the crystal structure of **1**_9_ proved difficult to obtain. Initial attempts yielded crystals that did not diffract better than 1.96 Å. We eventually used a racemic crystallographic approach.^43−45^ From the d enantiomer of **1**, d-**1**_9_ was produced, and it was purified and mixed with l-**1**_9_ to produce a racemic mixture. Crystals obtained from this mixture diffracted at 1.15 Å and made it possible to solve the structure in the *P*1 space group. The asymmetric unit contained the two enantiomeric macrocycles, though not related by a crystallographic inversion or glide plane, a feature also observed in the racemic crystals of proteins. The structure of the 9mer reveals a new fold (Figure 3). This fold differs from the 15mer, 16mer, and 23mer previously characterized with monomers bearing different side chains than that of **1**.^39,40^ As in these larger foldamers, the 9mer possesses a compact hydrophobic core comprised of dimercaptobenzene units. In the structure, three phenyl rings in positions *i*, *i*+2, and *i*+4 are stacked on top of each other, and this motif is repeated three times, giving an overall triangular shape (Figure 3a). This arrangement resembles that of the previously reported 15mer, formed from dimercaptobenzene appended with an amino acid and a nucleobase, which exhibited a similar stack of three aromatics but repeated five times in the structure (Figure 3c).^40^ The similarities between these structures suggest that the stacking of aromatic rings in the *i*, *i*+2, and *i*+4 positions is a recurrent arrangement amounting to a sort of secondary structure motif in this class of disulfide foldamers. However, while the 15mer structure admitted a pseudo-*C*_5_ symmetry axis to generate a symmetrical pentagon with five groups of three inequivalent rings, the structure of **1**_9_ adopts no (pseudo)symmetry. Careful examination of the stereochemistry of the disulfide bonds along the macrocycle shows no repeat motif (Figure 3b), indicating that all nine rings are in different environments. One can nevertheless envisage that the *C*_3_-symmetrical conformer of **1**_9_ exists in solution, possibly along with other stereochemical variations, and that these possible internal dynamics explain the broad NMR spectra. The side chains of the structure of **1**_9_ were heavily disordered and do not allow us to provide an interpretation of the stability of this fold at higher pH.

**Figure 4:**
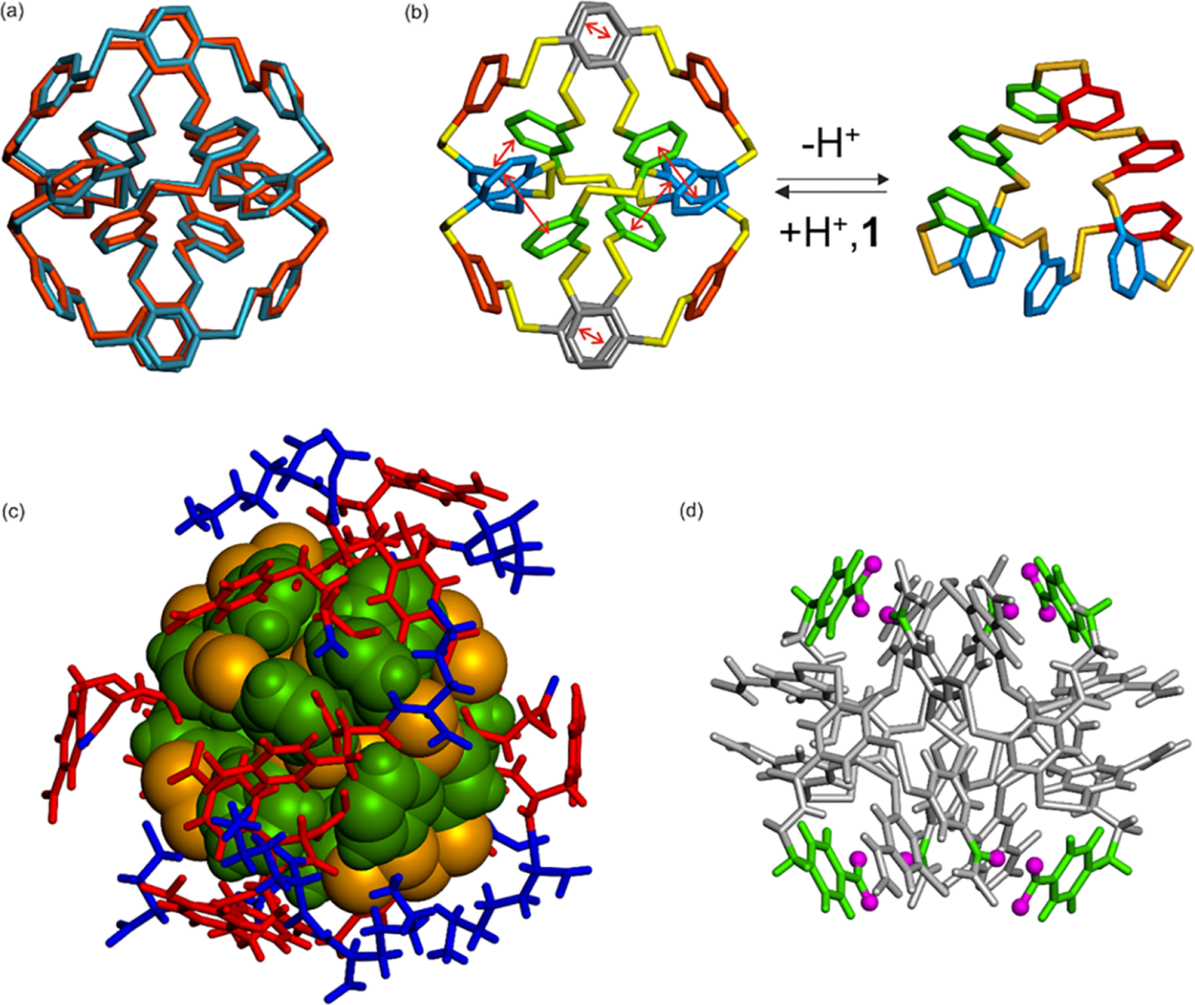
Crystal structure of **1**_16_. (a) Superimposition of the hydrophobic dimercaptobenzene core of **1**_16_ and that of a previously published 16mer made from a dithiol building block appended with a Phe(4-guanidinium)-Lys-OH side group.^39^ Hydrogen atoms have been omitted for clarity. (b) Scheme illustrating the radical structural and constitutional reversible change between **1**_16_ and **1**_9_. (c) Complete view of **1**_16_. The core is shown in space-filling representation in green (benzene rings) and gold (disulfide bonds). Side chains are shown in red (Phe(4-COOH) residues) and blue (Lys residues). (d) Tube representation of **1**_16_ with the hydrophobic core shown in gray. Side chains have all been removed except for equivalent Phe(4-COOH) residues (in green) whose carboxyl oxygen atoms point toward the carbonyl oxygen atom of a benzamide unit (shown as purple spheres).

In contrast, the crystal structure of **1**_16_ could be solved with most side chains visible in the final model, including the more flexible lysine side chains. Crystals of **1**_16_ diffracted at 1.06 Å, and racemic crystallography was not needed in this case. The asymmetric unit (again in the *P*1 lattice) contains two almost superimposable objects. The disulfide-linked aromatic core adopts a conformation that is essentially identical to that observed in another 16mer formed from another dithiol building block, appended with a Phe(4-guanidinium)-Lys-OH side group (Figure 4a).^39^ The phenyl rings all sit in an aromatic box with edge-to-face and face-to-face π-stacking interactions, which contribute to the structural stability of **1**_16_. However, the *i*, *i*+2, *i*+4 motif is absent in this fold. The fact that dissimilar dipeptide side groups can still yield similar folds suggests that folding is to a large degree driven by the conformational preferences of the hydrophobic core of disulfide-linked benzene units. At the same time, the system can be directed to form an altogether different ring size by changing (the degree of protonation of) the side groups. So it appears that the overall ring size is dictated by an interplay between side group interactions and how a given core allows side groups to be arranged. Figure 4b illustrates the extent of the structural and constitutional rearrangement that takes places between **1**_16_ and **1**_9_.

**Figure 5:**
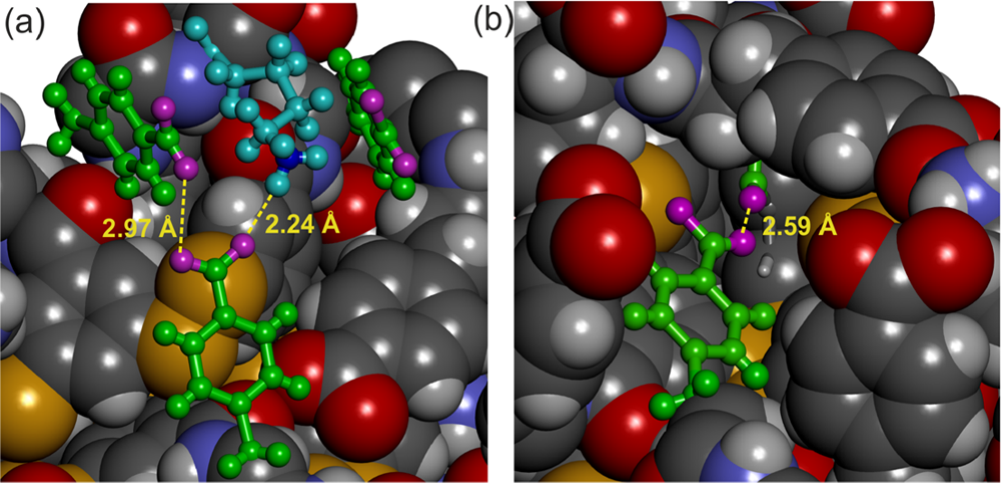
Specific interactions in the crystal structure of **1**_16_. Enlarged views of Phe(4-COOH) side chains presumed to exist mostly in their protonated form up to pH 7.2. Atoms are shown in space-filling representation except the groups of interest, which are shown in ball-and-stick representation with side chain Phe(4-COOH) and Lys side chains in green and cyan, respectively. Carboxylate oxygen atoms are shown in purple. Some noteworthy distances are indicated.

In the structure of **1**_16_, the dipeptide units also adopt well-defined conformations and shield the hydrophobic core from contact with water (Figure 4c). Notably, four Phe(4-COOH) aryl carboxylic acid groups buried at the surface of the hydrophobic core point toward the carbonyl groups of benzamide units at such a distance that interactions would be strongly repulsive and destabilizing if they were deprotonated, i.e., in their carboxylate form (Figures 4d and 5b). Presumably, these carboxylic acids are in their protonated form, although protons are not directly assignable in the electron density map, and form hydrogen bonds with the carbonyls. We speculate that, due to these intramolecular hydrogen bonds, deprotonation of these aryl-COOH groups becomes more difficult (leading to an elevated p*K*_a_, as observed experimentally; *vide supra*). However, when, upon raising the pH, these groups become deprotonated, the resulting charge repulsion between the carboxylate residues and the nearby amide carbonyl oxygens destabilizes **1**_16_. This effect is likely a contributor to the constitutional switching between **1**_16_ and **1**_9_. These four interactions are found at symmetrical positions in the structure of **1**_16_ (Figure 4d), which suggests that they are likely to contribute significantly to stabilizing the foldamer structure. A fifth short COO···O contact (thus presumed to be COOH···O) is shown in Figure 5b and may also contribute to the stability of **1**_16_. However, this contact also reflects the absence of symmetry of the overall structure and that some interactions between the dipeptide appendages may form and dissociate in a dynamic fashion in solution. Mirror effects may exist in **1**_9_, e.g., hydrogen bonds between carboxylates and amide NHs or lysine ammonium groups that would be destabilizing at lower pH upon protonation of the carboxyl group, but the low quality of the structure did not allow for their identification.

### pH Dependence of Foldamer Formation for Building Blocks **2**–**4**

To further investigate the role of building block structure in pH-dependent foldamer formation, other related building blocks **2**–**4** were synthesized and analyzed as shown in Table 1 and Figures S15–S26. Elongating the sequence of **1** with a histidine residue (building block **2**) yielded similar pH-dependent foldamer formation, that is, producing a 16mer and a 9mer at pH 6.0 and pH 8.2, respectively. Substituting the C-terminal lysine of **1** with serine-histidine produced building block **3**, which forms a 16mer or a tetramer, depending on the pH of the solution. The same was observed for monomer **4**, an analogue of **1** terminated with a primary amide instead of a carboxyl group. These results suggest that both the carboxylate terminus and lysine residues play roles in the stabilization of 9mers, although this role could not be identified in the structure of **1**_9_.

**Table 1:**
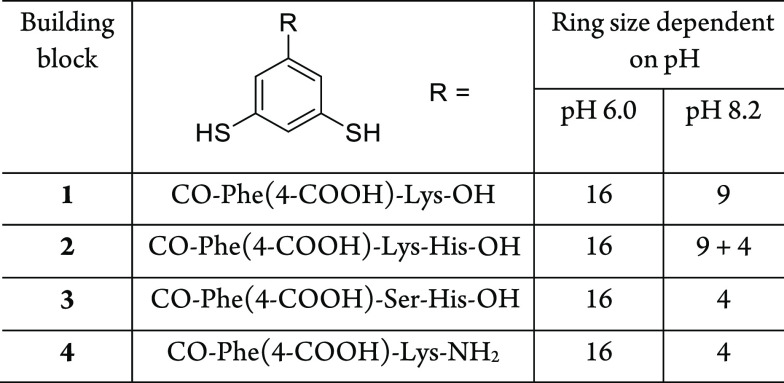
Main Products in Dynamic Combinatorial Libraries (DCLs) Prepared from Different Building Blocks at Different pHs

## Conclusions

Folding of oligomeric molecules with ionizable groups can be expected to be sensitive to pH, particularly when these groups are brought into close proximity upon folding, or when they are directly involved in non-covalent interactions. Such sensitivity normally results in pH-induced unfolding. For foldamers that feature dynamic covalent bonds, additional degrees of freedom are provided and other folded states are, in principle, accessible upon a reconfiguration of covalent bonds. Thus, instead of unfolding upon changing the ionization state of the molecule, the system may choose to populate an altogether different foldamer. Indeed, we observed such adaptive behavior in a dynamic combinatorial library (DCL) made from building block **1**, which, upon oxidation, formed a folded 16-membered ring at pH 6.0. Titrations suggest that the p*K*_a_ values of a subset of carboxylic acid groups in this foldamer are substantially perturbed (p*K*_a_ = 7–7.5), whereas carboxylic acids typically have p*K*_a_s of around 4.2–4.7. Thus, in the folded 16mer at pH 6.0, these groups are mostly protonated. Upon raising the pH to 8.2, the extent of deprotonation increases, and with that also the charge repulsion, which contributes to a structural reconfiguration, leading to the formation of a 9-membered foldamer. The pH-induced switching between these two foldamers is fully reversible, provided a sufficient amount of thiolate is present, and occurs over a remarkably narrow pH range, suggesting that folding is a cooperative process. The crystal structure of **1**_9_ revealed a new fold with nine inequivalent rings. The structure of **1**_16_ showed specific interactions involving ionizable functional groups of the side chains that shed light on its higher stability at lower pH. These results extend our previous report that DCLs made from building block **1** can also give rise to 12- and 13-membered foldamers, along with small amounts of a 14mer, upon templating with a suitable guest.^39^

Taken together, these observations illustrate the remarkable richness of the behavior of DCLs made from this particular building block, allowing as many as five distinct folded structures to be accessed. We attribute this uniquely diverse foldamer space to the frustration in the structure of this building block, which seems incapable of finding a single highly stable fold in which it maximizes attractive and minimizes repulsive interactions. Instead, the system seems to navigate a relatively shallow energy landscape, where different folded conformations differ only relatively little in stability. Small perturbations are then sufficient to shift the product distribution from one conformation and ring size to another, exemplified by the switchover from one foldamer to another by changing the pH by less than half a pH unit.

The newly discovered structures provide important additional entries into a growing class of macrocyclic disulfide foldamers that exhibit unusual structural complexity. After previous reports of the crystal structures of a 15mer,^40^ a 16mer, and a 23mer,^39^ we now add a 9mer and a second 16mer. Further populating this class of structures should reveal the rules and, with that, design principles behind this exciting new class of folded molecules. Progress along these lines is being made and will be reported in due course.

## Supporting Information Available

Experimental details, synthesis procedures, spectroscopic data, and crystallographic data (PDF)
