# Supplementary material for: (Re-)Directing Oligomerization of a Single Building Block into Two Specific Dynamic Covalent Foldamers through pH
Source: J Am Chem Soc. 2023 Jan 27;145(5):2822–9. doi: 10.1021/jacs.2c09325 (PMC9912251; doi:10.1021/jacs.2c09325)
Supplement: Supplementary file 1 — ja2c09325_si_001.pdf [file ja2c09325_si_001.pdf]

Supporting Information

(Re-)Directing Oligomerization of a Single Building Block into Two Specific Dynamic Covalent Foldamers through pH

Yulong Jin,[1,2] *Pradeep K. Mandal,*[3] *Juntian Wu,*[2] *Niklas Böcher,*[3] *Ivan Huc*[3,*] *and Sijbren Otto*[2,*]

[1]Beijing National Laboratory for Molecular Sciences, CAS Key Laboratory of Analytical Chemistry for Living Biosystems, Institute of Chemistry, Chinese Academy of Sciences, 100190 Beijing, China.

[2]Centre for Systems Chemistry, Stratingh Institute, Nijenborgh 4, 9747 AG, Groningen, the Netherlands.

[3]Department of Pharmacy and Center for Integrated Protein Science, Ludwig-Maximilians Universität, 81377 Munich, Germany.

*Email: ivan.huc@cup.lmu.de;

*Email: s.otto@rug.nl

Table of Contents

1. Experimental section S3
 1.1 General procedures S3
 1.2 Peptide synthesis S3
 1.3 Library preparation S5
 1.4 UHPLC analysis S6
 1.5 UHPLC-MS analysis S6
 1.6 CD spectroscopy S7
 1.7 pH switching cycles S7
 1.8 LC-IM-MS S7
 1.9 pH titration S8
 1.10 Crystallization of $\mathbf{1}_9$ and $\mathbf{1}_{16}$ S9
 1.11 Data collection and structure determination of L/D-$\mathbf{1}_9$ S9
 1.12 Data collection and structure determination of L-$\mathbf{1}_{16}$ S10
 1.13 CheckCIF validation of L/D-$\mathbf{1}_9$: S11
 1.14 CheckCIF validation of L-$\mathbf{1}_{16}$: S12
2. Supporting tables and figures S14
3. References S45

1. Experimental section

1.1 General procedures.

Wang resin and Fmoc-amino acid-Wang resins were purchased from Novabiochem, Fmoc-D-Lys(Boc)-OH was purchased from Iris Biotech, Fmoc-L-Phe(4-COOtBu)-OH was purchased from Chem-Impex International Inc and Fmoc-D-Phe(4-COOtBu)-OH was purchased from BLDpharm. 3,5-Bis(tritylthio)benzoic acid was synthesized via a previously reported procedure.[1] Rink Amide AM resin, Fmoc-L-Lys(Boc)-OH, Fmoc-L-Ser(tBu)-OH and 2-(1H-Benzotriazole-1-yl)-1,1,3,3-tetramethyluronium hexafluorophosphate (HBTU) were all purchased from GL Biochem (Shanghai, China). 1-Hydroxybenzotriazole hydrate (HOBT•H_2O) was obtained from Siam (Chicago, USA). Dimethylformamide (DMF) and acetonitrile (HPLC grade) were purchased from Fisher Scientific (Hampton, USA). Dichloromethane (DCM), piperidine, 4-methylmorpholine (NMM), diethyl ether, HCl, disodium hydrogen phosphate dodecahydrate ($Na_2HPO_4 \cdot 12H_2O$) and sodium dihydrogen phosphate dihydrate ($NaH_2PO_4 \cdot 2H_2O$) were all purchased from Beijing Chemical Works (Beijing, China). 1,2-Ethanedithiol (EDT), triisoproyl silane (TIS), and trifluoroacetic acid (TFA) were obtained from Sigma-Aldrich. Ultrapure water from a Milli Q water purification system (Burlington, USA) was used throughout. Other materials used for synthesis were commercially available and used as received. NMR spectra were recorded on Bruker Avance 600 MHz spectrometers. HRMS spectra were recorded on an Orbitrap Fusion Lumos Tribrid Mass Spectrometer.

1.2 Peptide synthesis

The synthesis of building blocks were performed using Fmoc solid-phase peptide synthesis on Wang resin (for building block **1**, **2**, and **3**) or Rink Amide AM resin (for building block **4**). Specifically, for the synthesis of L-peptide acids, pre-loaded Fmoc-amino acid-Wang resins were

used. For the synthesis of D-**1**, Wang resin was brominated and loaded with Fmoc-D-Lys(Boc)-OH according to a standard protocol.[2] Fmoc deprotection steps were carried out with 20% piperidine in DMF (2×5 min). In the coupling of subsequent amino acids or 3,5-bis(tritylthio)benzoic acid, 3-fold Fmoc-protected amino acids or 3,5-bis(tritylthio)benzoic acid in the presence of 3-fold HBTU, 3-fold HOBT and 8-fold NMM were used. Deprotection from the resin and removal of the protecting groups was performed using a cocktail of 94% TFA, 2.5% EDT, 2.5% water and 1% TIS for 2 h. The solution was then filtered, the filtrate was vacuum-dried with rotary evaporation and the residue was treated with cold diethyl ether. The white precipitate was collected by centrifugation and washed with cold diethyl ether three times. HPLC purifications were performed using water (eluent A) and acetonitrile (eluent B), which each contained 0.1% v/v trifluoroacetic acid as the modifier. The HPLC purification of building block L-**1**, **2**, **3**, **4** and the macrocycles L-$\mathbf{1}_4$, L-$\mathbf{1}_9$ and L-$\mathbf{1}_{16}$ was performed on a Shimadzu LC-20AR HPLC system with an Ultimate XB-C18 column (Welch, 21.2×250 mm, 5 μm). The HPLC purification of building block D-**1** and the macrocycles D-$\mathbf{1}_9$ and L-$\mathbf{1}_9$ was performed on a modular Waters preparative HPLC system (2545 Quaternary Gradient Module, 2489 UV/Vis Detector, 2707 Autosampler, Fraction Collector III) equipped with a NUCLEODUR C18 HTec column (Macherey-Nagel, 21×125 mm, 5 μm). The building blocks and cyclic compounds were obtained at a purity higher than 95%. Salt exchange from TFA to HCl was performed twice by treating purified macrocycles with excess 0.1 M HCl and subsequent freeze-drying.

Building block **1**, ^{1}H NMR (600 MHz, D_2O, 298K, shown in Figure S1a) δ (ppm), 7.84 (d, *J* = 8.2 Hz, 2H), 7.32 (d, *J* = 8.1 Hz, 2H), 7.22 (s, 1H), 7.12 (d, *J* = 1.7 Hz, 2H), 4.75 (dd, *J* = 8.8, 6.8 Hz, 1H), 4.25 (dd, *J* = 8.9, 5.1 Hz, 1H), 3.23 (dd, *J* = 13.8, 6.7 Hz, 1H), 3.10 (dd, *J* = 13.8, 8.9 Hz, 1H),

2.85 (t, J = 7.6 Hz, 2H), 1.78 (m, 1H), 1.64 (m, 1H), 1.56 (m, 2H), 1.29 (m, 2H). MALDI-FTICR-MS, monoisotopic m/z calculated for $C_{23}H_{27}N_3O_6S_2$ $[M+H]^+$: 506.1414, observed: 506.1416.

Building block **2**, ^1H NMR (600 MHz, D_2O, 298K, shown in Figure S1b) δ 8.48 (1H), 7.83 (d, J = 8.0 Hz, 2H), 7.33-7.29 (m, 3H), 7.16 (m, 2H), 4.73 (m, 1H), 4.48 (dd, J = 8.0, 5.3 Hz, 1H), 4.18 (dd, J = 8.7, 5.7 Hz, 1H), 3.22-3.14 (m, 2H), 3.11 (m, 1H), 3.03 (m, 1H), 2.85 (t, J = 7.7 Hz, 2H), 1.75-1.67 (m, 1H), 1.65-1.58 (m, 1H), 1.54 (m, 2H), 1.23 (m, 2H). MALDI-FTICR-MS, monoisotopic m/z calculated for $C_{29}H_{34}N_6O_7S_2$ $[M+H]^+$: 643.2003, observed: 643.2002.

Building block **3**, ^1H NMR (600 MHz, D_2O, 298K, shown in Figure S1c) δ 8.49 (1H), 7.86 (2H), 7.34 (3H), 7.16 (2H), 4.80 (1H), 4.60 (1H), 4.35 (1H), 3.75 (2H), 3.24 (2H), 3.10 (2H). MALDI-FTICR-MS, monoisotopic m/z calculated for $C_{26}H_{27}N_5O_8S_2$ $[M+H]^+$: 602.1374, observed: 602.1373.

Building block **4**, ^1H NMR (600 MHz, D_2O, 298K, shown in Figure S1d) δ (ppm) 7.86 (d, J = 8.3 Hz, 2H), 7.32 (d, J = 8.1 Hz, 2H), 7.27 (s, 1H), 7.16 (d, J = 1.7 Hz, 2H), 4.74-4.71 (m, 1H), 4.19 (dd, J = 9.1, 5.4 Hz, 1H), 3.21 (dd, J = 13.7, 7.4 Hz, 1H), 3.13 (dd, J = 13.7, 8.5 Hz, 1H), 2.85 (t, J = 7.6 Hz, 2H), 1.72 (m, 1H), 1.61 (m, 1H), 1.55 (m, 2H), 1.29 (m, 2H). MALDI-FTICR-MS, monoisotopic m/z calculated for $C_{23}H_{28}N_4O_5S_2$ $[M+H]^+$: 505.1574, observed: 505.1572.

1.3 Library preparation

Building blocks (final concentration 1.0 mM) were dissolved in phosphate buffer (25 mM or 50 mM). All the libraries were set up in an HPLC vial (12 × 32 mm) with a Teflon-coated screw cap. All the HPLC vials were equipped with a cylindrical stirrer bar (2 × 5 mm, Teflon coated) and stirred at 150 r.p.m. using an IKA RCT basic hot plate stirrer. All experiments were performed at 40 °C.

1.4 UHPLC analysis

UHPLC analyses were performed on a Shimadzu LC-40D XR UHPLC system. The separation systems were all equipped with a photodiode array detector set at a detection wavelength of 254 nm. Samples were analyzed on a HALO peptide ES-C18 column (160 Å, 2 μm, 2.1 × 150 mm), using water (eluent A) and acetonitrile (eluent B), which each contained 0.1% v/v trifluoroacetic acid as the modifier. A flow rate of 0.2 mL min^{-1} and a column oven temperature of 30 °C were applied. Gradient: 0-1-12-12.2-16 min, 5%-15%-60%-5%-5%B. Sample preparation was performed by diluting 5.0 μL of the library with 30 μL of doubly distilled water. HPLC injection volume is 5 μL.

1.5 LC-MS analysis

UHPLC-MS analyses (for dynamic combinatorial libraries (DCLs) made from building block **1**) were performed on a Thermo Scientific UltiMate 3000 UHPLC system coupled to a Thermo Scientific Fusion Lumos Obitrap mass spectrometer. Samples were analyzed on an Acquity UPLC Protein BEH C4 column (300 Å, 1.7 μm, 2.1 × 150 mm), using water (eluent A) and acetonitrile (eluent B), which each contained 0.1% v/v formic acid as the modifier. A flow rate of 0.2 mL min^{-1} and a column oven temperature of 35 °C were applied. Gradient: 0-3-20-22 min, 5%-5%-70%-70% B. Sample preparation was performed by diluting 5.0 μL of the library with 30 μL of double distilled water. UHPLC injection volume was 3 μL. The mass spectrometer was operated in positive electrospray ionization mode with the ionization parameters: capillary voltage, 3 kV; sheath gas (Arb), 35; aux gas (Arb), 15; sweep gas (Arb), 2; ion transfer tube temperature, 280 °C; vaporizer temperature, 150 °C; orbitrap resolution, 120000; scan range (m/z), 200-2000; RF lens (%), 30; AGC target, $4.0e^5$; maximum injection time (ms), 50; microscan, 1; data type, profile.

UPLC-MS analysis (for DCLs made from building block **2** and **3**) were carried out on a Waters Acquity UPLC H-class system coupled to a Waters Xevo-G2 TOF mass spectrometer. DCLs made from building block **4** were analyzed on an UltiMate 3000 UHPLC system equipped with a diode-array detector and connected to an LCQ Fleet mass spectrometer.

1.6 CD spectroscopy

Spectra were recorded on a Jasco J-810 spectrometer with a Peltier temperature controller. Heat-cool cycles were applied from 20 to 90 °C in steps of 10 °C at a rate of 1 °C min^{-1} and maintained for 2 min at every temperature before measuring. Spectra were obtained as averages of three measurements from 200 to 400 nm with a scanning speed of 150 nm min^{-1} and a bandwidth of 1 nm. A quartz cuvette with a 1 cm path length was used for the measurements. The purified $\mathbf{1}_9$ and $\mathbf{1}_{16}$ were redissolved in 25 mM phosphate buffer at pH 8.2 and pH 6.0, respectively, for the temperature dependent CD measurements. The concentration of all samples was kept as 0.10 mM in building block.

1.7 pH switching cycles

The buffer exchange was performed using a 3K centrifugal filter (Amicon Ultra-0.5 mL). Briefly, the library solution was added onto the centrifugal filter which was centrifuged at 10,000 r/min for 10 min. The concentrated sample was washed twice with water and then diluted with phosphate buffer of a different pH. After one day, the library was analyzed by UHPLC.

1.8 LC-IM-MS

UPLC-Ion mobility MS measurement was performed on a Waters ACQUITY UPLC I-Class PLUS System coupled to a Waters Synapt XS high resolution mass spectrometer. Samples were analyzed on a HALO peptide ES-C18 column (160 Å, 2 µm, 2.1 × 150 mm), using water (eluent A) and acetonitrile (eluent B), which contained 0.1% v/v formic acid as the modifier. A flow rate of 0.2

mL min^{-1} and a column oven temperature of 35 °C were applied. Gradient: 0-3-20-22 min, 5%-5%-70%-70% B. Sample preparation was performed by diluting 5.0 µL of the library with 30 µL of double distilled water. UPLC injection volume was 3 µL. The parameters of the ion source were as follows: capillary voltage, +2.5 kV; sampling cone voltage, 40 V; source offset voltage, 4 V; source temperature, 120 °C; desolvation temperature, 20 °C; cone gas flow (nitrogen), 50 Lh^{-1}; desolvation gas flow (nitrogen), 800 Lh^{-1}; nebuliser, 6.5 bar. Ion mobility parameters were: 1.0 V trap DC entrance voltage, 2.0 V trap DC bias voltage, −2.0 V trap DC voltage, 0 V trap DC exit voltage, −20.0 V IMS DC entrance voltage, 1.0 V helium cell DC voltage, −20.0 V helium exit voltage, 2.0 V IMS bias voltage, 20.0 V IMS DC exit voltage, 5.0 V transfer DC entrance voltage, 15.0 V transfer DC exit voltage, 300 m s^{-1} trap wave velocity, 0.5 V trap wave height voltage, 300 m s^{-1} IMS wave velocity, 0.5 V IMS wave height voltage, 247 m s^{-1} transfer wave velocity and 0.2 V transfer wave height voltage.

1.9 pH titration

The purified samples were dissolved in water (final concentration 0.51 mM in building block, 1.5 mL) and added into a 2 mL polypropylene plastic tube containing a Teflon-coated magnetic stirring bar (5×2 mm). For the titration of monomer, tetramer and 9mer, 0.5 M HCl was first added to the solution step by step to lower the pH to ~2.7. The titration of the 16mer was started with a freshly prepared solution of 16mer in water without reducing the pH. Small aliquots (2 or 5 µL) of 0.02, 0.1 or 0.5 M NaOH solution were then added to the samples and the pH was monitored using a Mettler Toledo SevenCompact pH meter with an InLab® Ultra-Micro-ISM sensor. Every pH point was measured twice to confirm that the reading had stabilized. Every sample was titrated at least in duplicate.

1.10 Crystallization of $\mathbf{1}_9$ and $\mathbf{1}_{16}$

Aqueous solutions of L-$\mathbf{1}_9$ and D-$\mathbf{1}_9$ were prepared as HCl salt and dissolved using pure water to a final concentration of 25 mg/mL. Aqueous solutions of L-$\mathbf{1}_{16}$ were prepared by dissolving the lyophilized powder using pure water and 3 µL of 1 M HCl to a final concentration of 25 mg/mL. Racemic $\mathbf{1}_9$ was prepared by mixing the enantiopure solutions L-$\mathbf{1}_9$ and D-$\mathbf{1}_9$ 1:1 (monitored by UV). Crystallization trials of L/D-$\mathbf{1}_9$ and L-$\mathbf{1}_{16}$ were carried out using standard sitting drop vapor diffusion method at 293 K. X-ray quality crystals for L/D-$\mathbf{1}_9$ (Figure S27) were obtained after 4 days by addition of 1.2 µL of L/D-$\mathbf{1}_9$ and 1.2 µL of 30% v/v polyethylene glycol 400, 100 mM HEPES buffer (pH 7.5) and 200 mM calcium chloride from the reservoir solution. A single crystal was fished using micro loops and plunged into liquid nitrogen directly such that the mother liquor served as cryo-protectant. X-ray quality crystals for L-$\mathbf{1}_{16}$ (Figure S27) were obtained after 10 days by addition of 1.0 µL of L-$\mathbf{1}_{16}$ and 2.0 µL of 20% w/v polyethylene glycol 6000, 100 mM sodium acetate buffer (pH 5.0), 200 mM sodium chloride from the reservoir solution. Single crystals of L-$\mathbf{1}_{16}$ were fished using micro loops, quickly soaked in reservoir solution supplemented with 33% v/v glycerol and plunged into liquid nitrogen.

1.11 Data collection and structure determination of L/D-$\mathbf{1}_9$

The X-ray diffraction data was collected at the ID23-1 beamline[3] in the European Synchrotron Radiation Facility (ESRF), Grenoble. Diffraction data was measured at T = 100 K, $d$min = 1.15Å, λ = 0.6888Å. The crystal was exposed for 0.01 s and 0.2° oscillation per frame and a rotation pass of 360° was measured using a Dectris Pilatus 6M detector. Diffraction data was processed using the *autoPROC* pipeline.[4-8] The crystal belonged to the Triclinic space group $P1$ with unit cell parameters: a = 18.519 (1) Å, b = 25.301 (8) Å, c = 35.771 (12) Å, α = 70.152° (1), β = 88.272° (2), γ = 82.314° (2); V = 15621 (7) Å^3 and 2 molecules per asymmetric unit (Z = Z' = 2). The

structure was solved with the program *SHELX*[9] and refined by full-matrix least-squares method on F^2 with *SHELXL*-2014[10] within *Olex2*[11] (Figure S28). After each refinement step, visual inspection of the model and the electron-density maps were carried out using *Olex2*[11] and *Coot*.[12] The initial structure revealed most of the main-chain atoms of an L-$\mathbf{1}_9$ macrocycle. After several iterations of least-squares refinement the main-chain trace improved for a second macrocycle L-$\mathbf{1}_9$. All side chains of phenyl carboxylate and lysine were observed to be disordered and were either omitted or refined with partial occupancy and isotropic displacement parameters. AFIX, DFIX and FLAT instructions were used to improve the geometry of molecules. Constraints and restraints on anisotropic displacement parameters were implemented with EADP, DELU, SIMU, RIGU and ISOR instructions. After several attempts to model the disordered side chains, the SQUEEZE[13] procedure was used to flatten the electron density map. Very disordered side chains and solvent molecules were removed. Hydrogen atoms were not added due to the poor quality of the data.

1.12 Data collection and structure determination of L-$\mathbf{1}_{16}$

The X-ray diffraction data was collected at the P13 beamline[14] operated by EMBL Hamburg, at the PETRA III storage ring (DESY, Hamburg) with a Dectris Pilatus 6M detector. Diffraction data were measured at T = 100 K, $d$min = 1.15 Å, λ = 0.82656 Å. The crystal was exposed for 0.04 s and 0.15° oscillation per frame. 2800 images were collected in a sweep with a total exposure time of 112 s. Diffraction data was processed using the program *CrysAlis^{Pro}*.[15] The crystal belonged to the Triclinic space group *P*1 with unit cell parameters: a = 25.676 (7) Å, b = 28.927 (5) Å, c = 48.437 (9) Å, α = 104.581° (2), β = 98.692° (2), γ = 95.045° (2); V = 34119.3 (13) Å^3 and 2 molecules per asymmetric unit (Z = Z' = 2). The structure was solved with the program *SHELXT*[9] and refined by full-matrix least-squares method on F^2 with *SHELXL*-2014[10] within *Olex2*[11] (Figure S29). The initial structure revealed all main-chain atoms and several side chains of two L-$\mathbf{1}_{16}$

macrocycles. After each refinement step, visual inspection of the model and the electron-density maps were carried out using *Olex2*[11] and *Coot*.[12] Some side chains of phenyl carboxylate and lysine were observed to be disordered and were either omitted or refined with partial occupancy and isotropic displacement parameters. AFIX, DFIX and FLAT instructions were used to improve the geometry of molecules. Constraints and restraints on anisotropic displacement parameters were implemented with EADP, DELU, SIMU, RIGU and ISOR instructions. After several attempts to model the disordered side chains, the SQUEEZE[13] procedure was used to flatten the electron density map. Very disordered side chains and solvent molecules were removed. Hydrogen atoms were placed at idealized positions except for those at disordered/missing side chains.

Statistics of data collection and refinement are described in Table S1. The final cif files were checked using IUCr's *checkCIF* algorithm. Due to large volume fractions of disordered solvent molecules, weak diffraction intensity and poor resolution, a number of A- and B-level remain in the *checkCIF* file. These alerts are inherent to the data and refinement procedures. They are listed below and have been divided into two groups. The first group illustrates weak quality of the data and refinement statistics if compared to that expected for small molecule structures from highly diffracting crystals. The second group is connected to decisions made during refinement and explained below. Atomic coordinates and structure factors for L/D-$\mathbf{1}_9$ and L-$\mathbf{1}_{16}$ were deposited in the Cambridge Crystallographic Data Centre (CCDC) with accession codes 2183369 and 2183384 respectively. The data is available free of charge upon request (www.ccdc.cam.ac.uk/).

1.13 CheckCIF validation of L/D-$\mathbf{1}_9$:

Group 1 (these alerts illustrate weak quality of the data and refinement statistics if compared to that expected for small molecule structures from highly diffracting crystals):

THETM01_ALERT_3_A The value of sine(theta_max)/wavelength is less than 0.550

Calculated sin(theta_max)/wavelength =   0.4348
PLAT029_ALERT_3_A _diffrn_measured_fraction_theta_full value Low .   0.863 Why?
PLAT082_ALERT_2_A High R1 Value .... 0.27 Report
PLAT084_ALERT_3_A High wR2 Value (i.e. > 0.25) .... 0.60 Report
PLAT242_ALERT_2_B Low 'MainMol' Ueq as Compared to Neighbors of N0AA Check
PLAT242_ALERT_2_B Low 'MainMol' Ueq as Compared to Neighbors of N278 Check
PLAT306_ALERT_2_B Isolated Oxygen Atom (H-atoms Missing ?) .... O4 Check
PLAT340_ALERT_3_A Low Bond Precision on C-C Bonds .... 0.06866 Ang
PLAT911_ALERT_3_B Missing FCF Refl Between Thmin & STh/L= 0.435 2953 Report

Group 2 alert (are connected with decision made during refinement and explained below):

PLAT097_ALERT_2_B Large Reported Max. (Positive) Residual Density 1.95 eA-3

This positive residual density corresponds to an S atom (S18A) that was anisotropically refined. The peak remained despite attempts to improve geometry and temperature parameters.

1.14 CheckCIF validation of L-1$_{16}$:

Group 1 (these alerts illustrate weak quality of the data and refinement statistics if compared to that expected for small molecule structures from highly diffracting crystals):

THETM01_ALERT_3_A The value of sine(theta_max)/wavelength is less than 0.550
Calculated sin(theta_max)/wavelength =   0.4717
PLAT029_ALERT_3_A_diffrn_measured_fraction_theta_full value Low .   0.933 Why?
PLAT035_ALERT_1_B_chemical_absolute_configuration Info Not Given Please Do !
PLAT084_ALERT_3_B_High wR2 Value (i.e. > 0.25) .... 0.37 Report
PLAT241_ALERT_2_A_High 'MainMol' Ueq as Compared to Neighbors of C80 Check
PLAT316_ALERT_2_A_Too many H on C in C=N Moiety in Main Residue .. C96 Check
PLAT306_ALERT_2_B_Isolated Oxygen Atom (H-atoms Missing ?) .... O3AA Check
PLAT315_ALERT_2_B_Singly Bonded Carbon Detected (H-atoms Missing). C25 Check
PLAT414_ALERT_2_A_Short Intra D-H..H-X H48B ..H395 . 1.71 Ang.
PLAT250_ALERT_2_B_Large U3/U1 Ratio for Average U(i,j) Tensor .... 4.7 Note
PLAT340_ALERT_3_B_Low Bond Precision on C-C Bonds .... 0.02613 Ang
PLAT911_ALERT_3_B_Missing FCF Refl Between Thmin & STh/L= 0.472 3655 Report

Group 2 alert (are connected with decision made during refinement and explained below):

SHFSU01_ALERT_2_A The absolute value of parameter shift to su ratio > 0.20
Absolute value of the parameter shift to su ratio given 0.581
Additional refinement cycles did not improve this.
PLAT080_ALERT_2_A Maximum Shift/Error 0.58 Why ?
Additional refinement cycles did not improve this.
PLAT201_ALERT_2_A Isotropic non-H Atoms in Main Residue(s) 302 Report
O4AA O3 O4 O2BA O0EA etc.

These belong to the disordered peptide sidechains that were refined with isotropic displacement parameters.

PLAT202_ALERT_3_A Isotropic non-H Atoms in Anion/Solvent 237 Check

O8KA O2 O2E O1BA O3B O4BA etc.

These belong to the disordered peptide sidechains that were refined with isotropic displacement parameters.

2. Supporting tables and figures

Table S1 Crystallographic data and refinement details for L/D-**1**$_9$ and L-**1**$_{16}$.

Foldamers	L/D-**1**$_9$	L-**1**$_{16}$
Empirical formula	$C_{103}N_{9.5}O_{23.25}S_{18}$	$C_{309.5}H_{247.5}N_{34.5}O_{73.5}S_{32}$
Formula weight	2319.21	6651.83
Temperature	100.15 K	100.15 K
Wavelength	0.6888 Å	0.8265 Å
Crystal system	Triclinic	Triclinic
Space group	*P*1	*P*1
Unit cell dimensions	*a* = 18.519 (1) Å<br>*b* = 25.301 (8) Å<br>*c* = 35.771 (12) Å<br>*α* = 70.152 (10)°<br>*β* = 88.272 (2)°<br>*γ* = 82.314 (7)°	*a* = 25.676 (7) Å<br>*b* = 28.927 (5) Å<br>*c* = 48.437 (9) Å<br>*α* = 104.581 (2)°<br>*β* = 98.692 (2)°<br>*γ* = 95.045 (2)°
Volume	15621.0 (7) Å^3	34119.3 (13) Å^3
Z	2	2
Density (calculated)	0.493 g/cm^3	0.647 g/cm^3
Absorption coefficient	0.139 μ/mm^{-1}	0.212 μ/mm^{-1}
Color and shape	Colorless, plates	Colorless, plates
Crystal size	0.050 x 0.050 x 0.002 mm	0.100 x 0.100 x 0.002 mm
Index ranges	-16 ≤ h ≤ 16<br>-21 ≤ k ≤ 21<br>-31 ≤ l ≤ 31	-24 ≤ h ≤ 24<br>-25 ≤ k ≤ 25<br>-45 ≤ l ≤ 45
Reflections collected	66300	232952
R_{int}	0.0895	0.0837
Data/restraints/parameters	36301/597/801	111383/1491/3050
Goodness-of-fit on F^2	1.749	1.211
Final R indexes [$I > 2\sigma(I)$]	R_1 = 0.2659<br>wR_2 = 0.5266	R_1 = 0.1366<br>wR_2 = 0.3220
Final R indexes [all data]	R_1 = 0.3607<br>wR_2 = 0.6041	R_1 = 0.1873<br>wR_2 = 0.3746
Largest diff. peak and hole	1.95/-1.49 e Å^{-3}	0.58/-0.60 e Å^{-3}
Total potential solvent accessible void volume from SQUEEZE	10836.7 Å^3	19068.2 Å^3
Electron count/cell	3507	6449
CCDC #	2183369	2183384

(a)

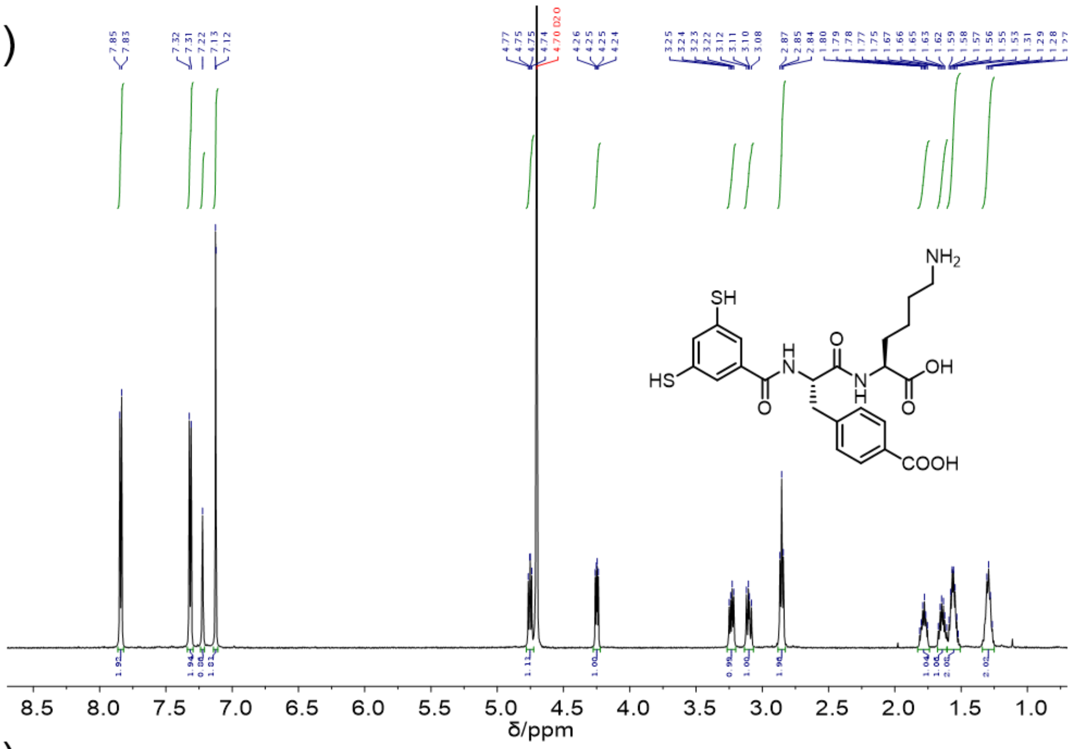

(b)

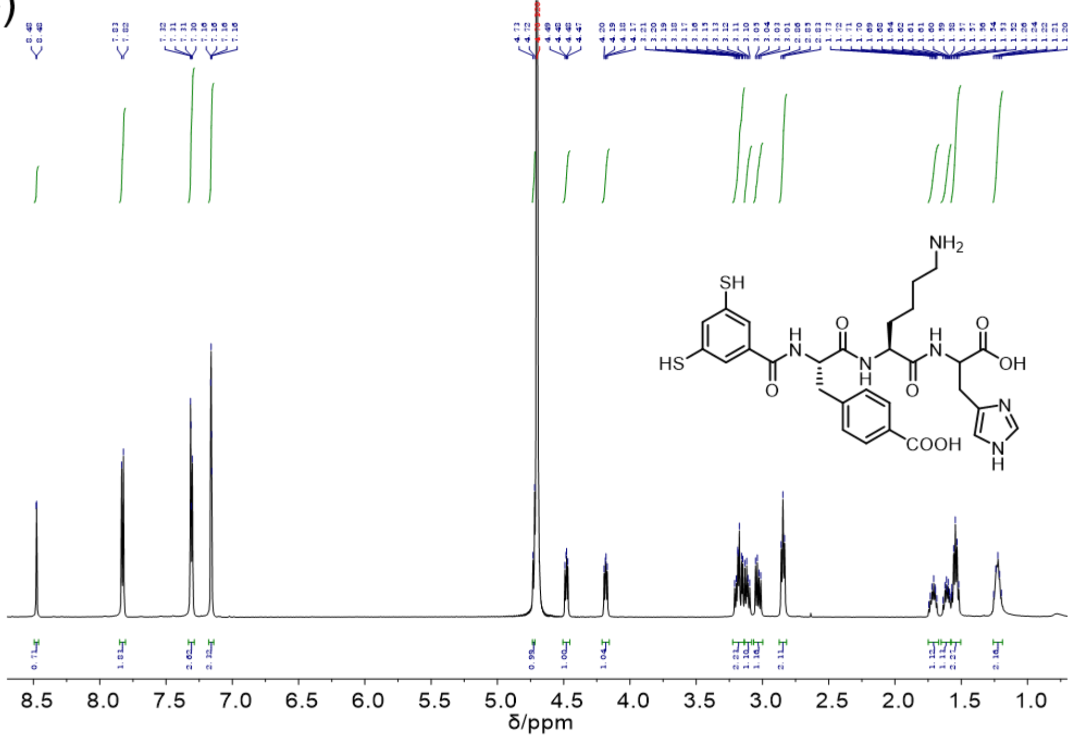

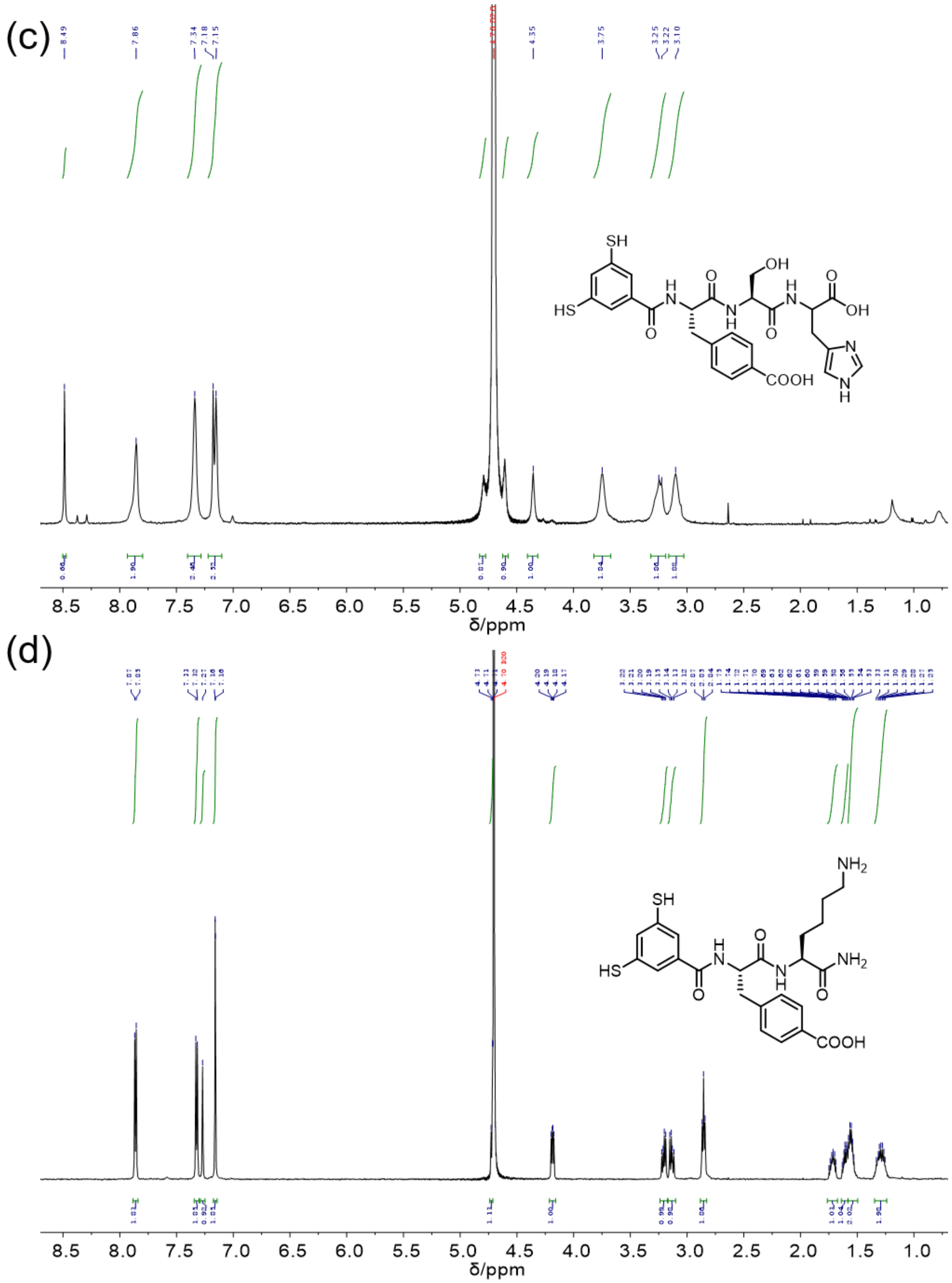

Figure S1. ^{1}H NMR spectra (600 MHz) at 298K of building block (a) **1**, (b) **2**, (c) **3**, and (d) **4** in D_2O.

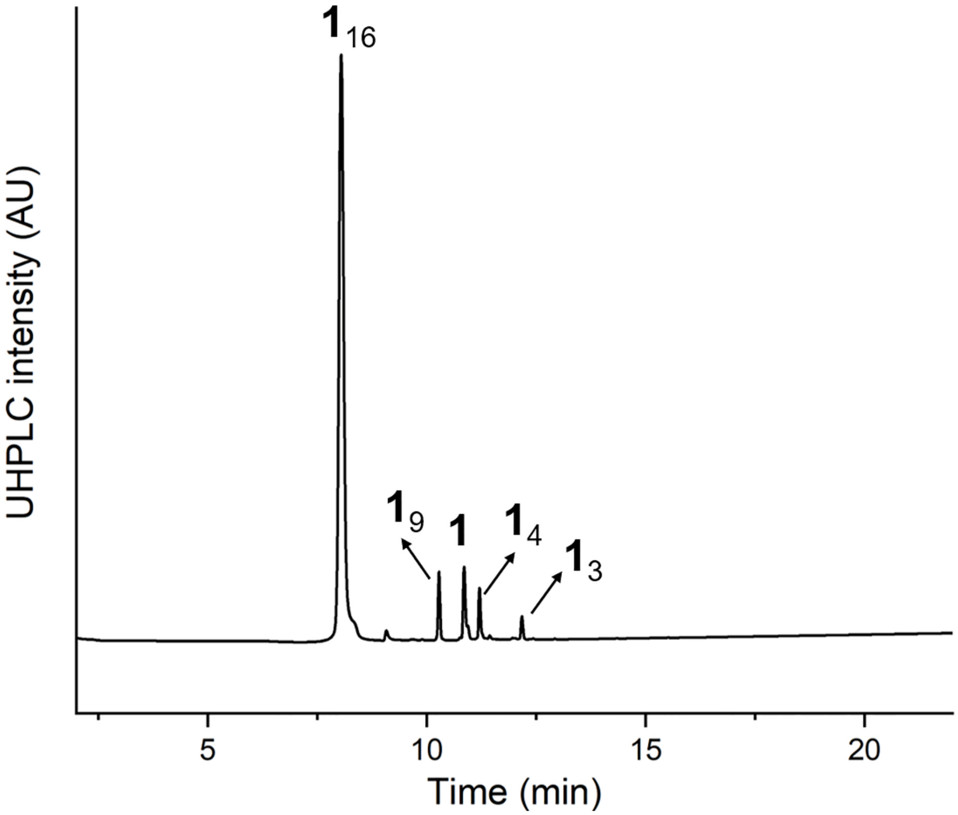

Figure S2. UHPLC-PDA-MS analyses of the DCL made from 1.0 mM building block **1** in 25 mM phosphate buffer (pH 6.0) at day 4. Wavelength: 254 nm. The retention times for different compounds are: 8.05 min ($\mathbf{1}_{16}$); 10.28 min ($\mathbf{1}_9$); 10.85 min (**1**); 11.21 min ($\mathbf{1}_4$); 12.17 min ($\mathbf{1}_3$).

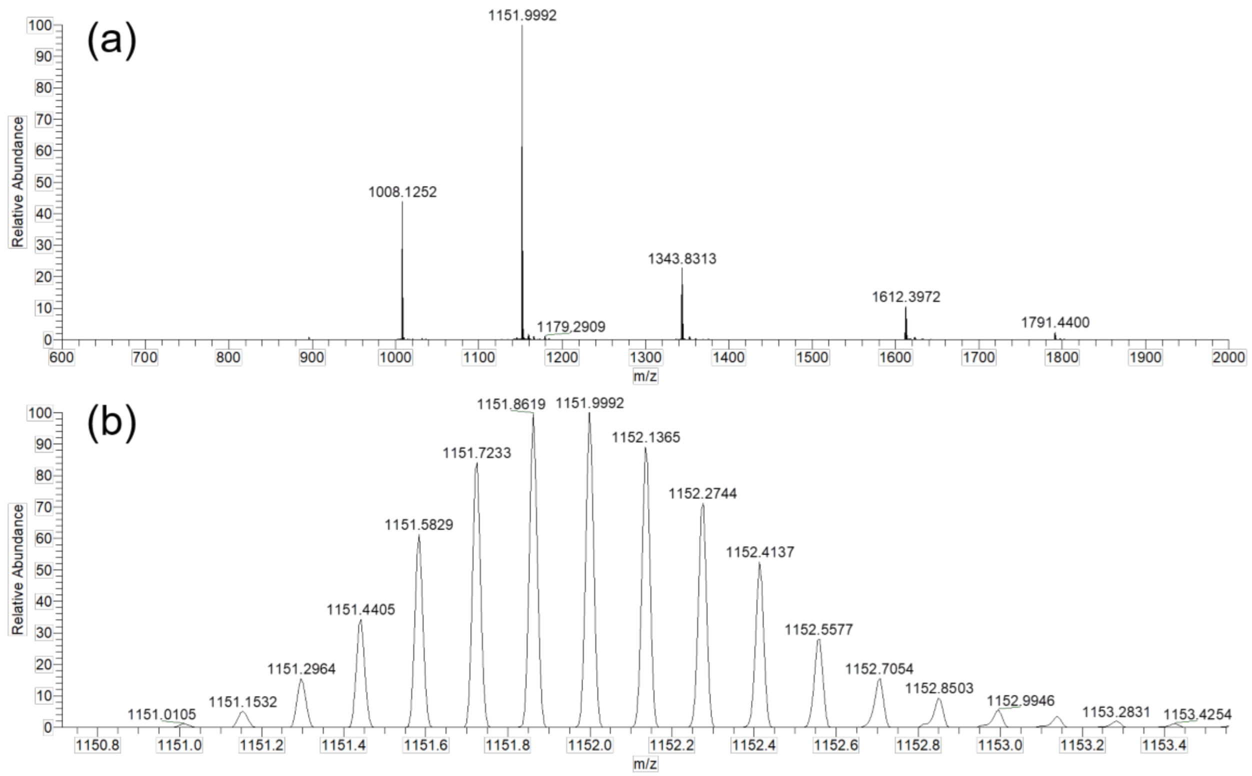

Figure S3. Mass spectrum of $\mathbf{1}_{16}$ extracted from the UHPLC-PDA-MS analysis shown in Figure S2. (a) Full mass spectrum: m/z observed: 1008.1252 $[M+8H]^{8+}$, 1151.9992 $[M+7H]^{7+}$, 1343.8313 $[M+6H]^{6+}$, 1612.3972 $[M+5H]^{5+}$. (b) Isotopic ion peaks of $[M+7H]^{7+}$. Monoisotopic m/z calculated: 1150.9924. Monoisotopic m/z observed: 1151.0105.

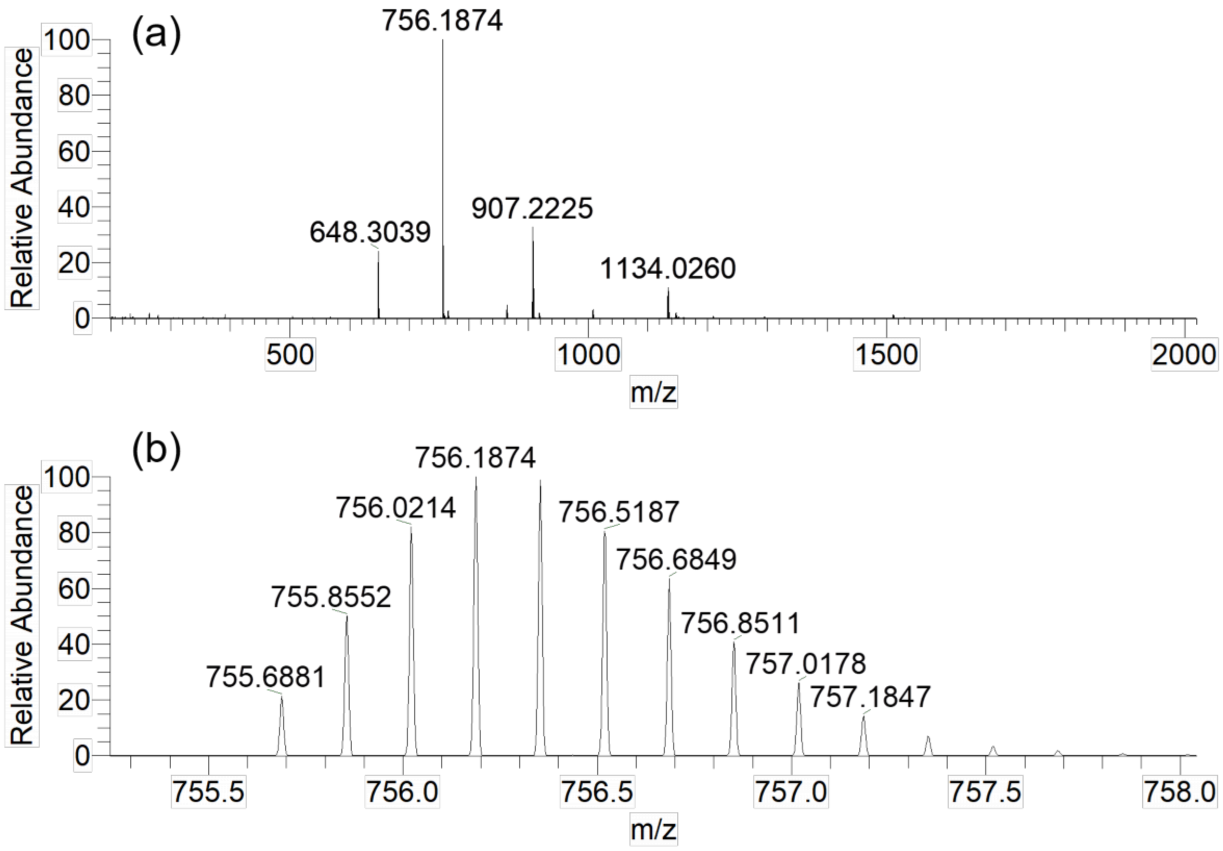

Figure S4. Mass spectrum of **1**$_9$ extracted from the UHPLC-PDA-MS analysis shown in Figure S2. (a) Full mass spectrum: m/z observed: 648.3039 $[M+7H]^{7+}$, 756.1874 $[M+6H]^{6+}$, 907.2225 $[M+5H]^{5+}$, 1134.0260 $[M+4H]^{4+}$. (b) Isotopic ion peaks of $[M+6H]^{6+}$. Monoisotopic m/z calculated: 755.6850. Monoisotopic m/z observed: 755.6881.

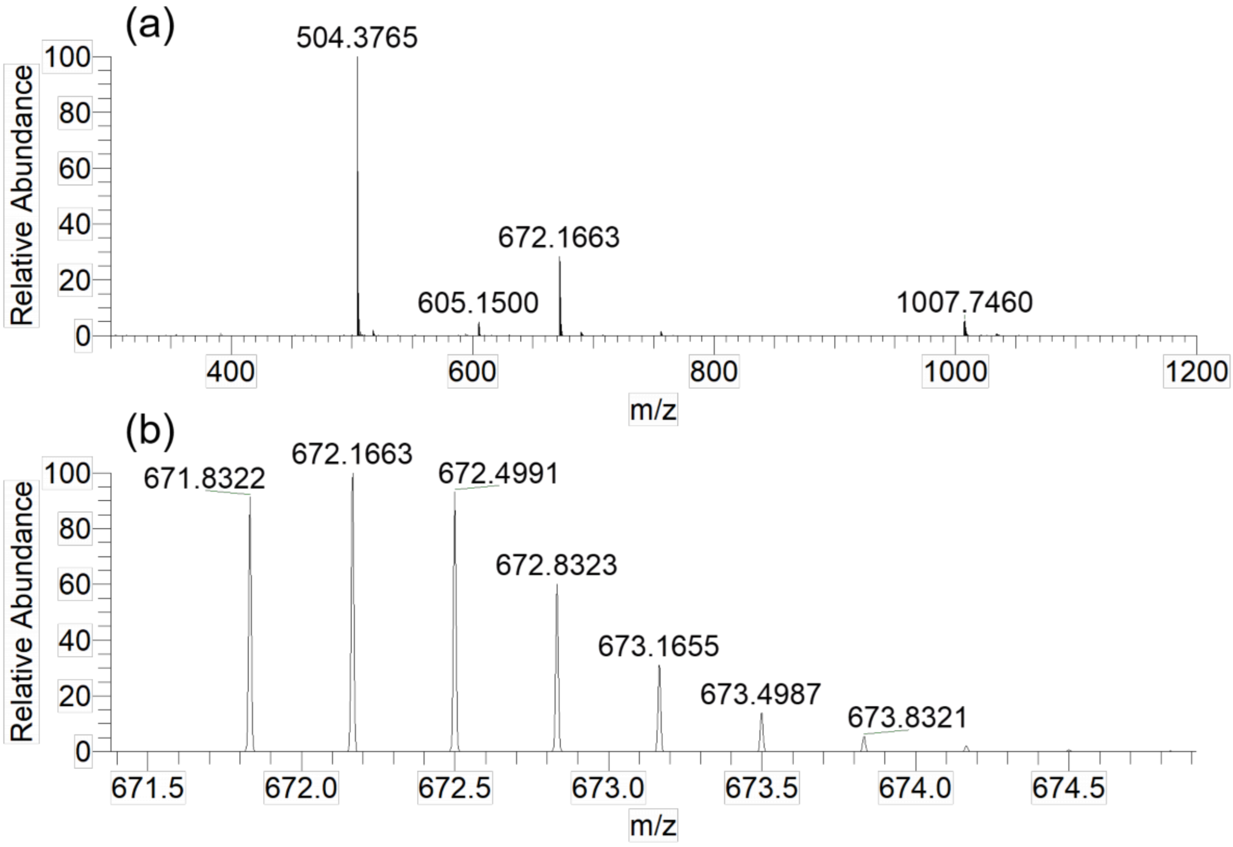

Figure S5. Mass spectrum of **1**$_4$ extracted from the UHPLC-PDA-MS analysis shown in Figure S2. (a) Full mass spectrum: m/z observed: 504.3765 $[M+4H]^{4+}$, 672.1663 $[M+3H]^{3+}$, 1007.7460 $[M+2H]^{2+}$. (b) Isotopic ion peaks of $[M+3H]^{3+}$. Monoisotopic m/z calculated: 671.8319. Monoisotopic m/z observed: 671.8322.

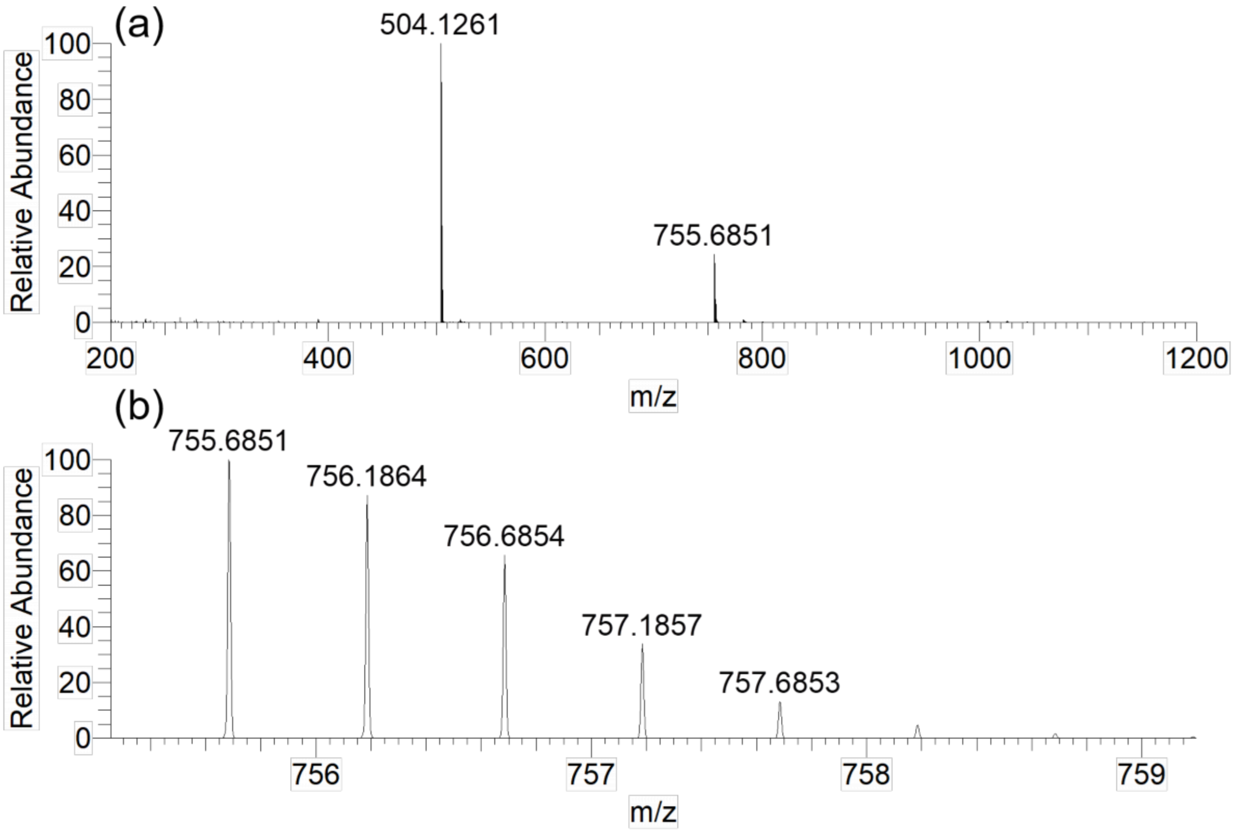

Figure S6. Mass spectrum of $\mathbf{1}_3$ extracted from the UHPLC-PDA-MS analyses shown in Figure S2. (a) Full mass spectrum: m/z observed: 504.1261 $[M+3H]^{3+}$, 755.6851 $[M+2H]^{2+}$. (b) Isotopic ion peaks of $[M+2H]^{2+}$. Monoisotopic m/z calculated: 755.6850. Monoisotopic m/z observed: 755.6851.

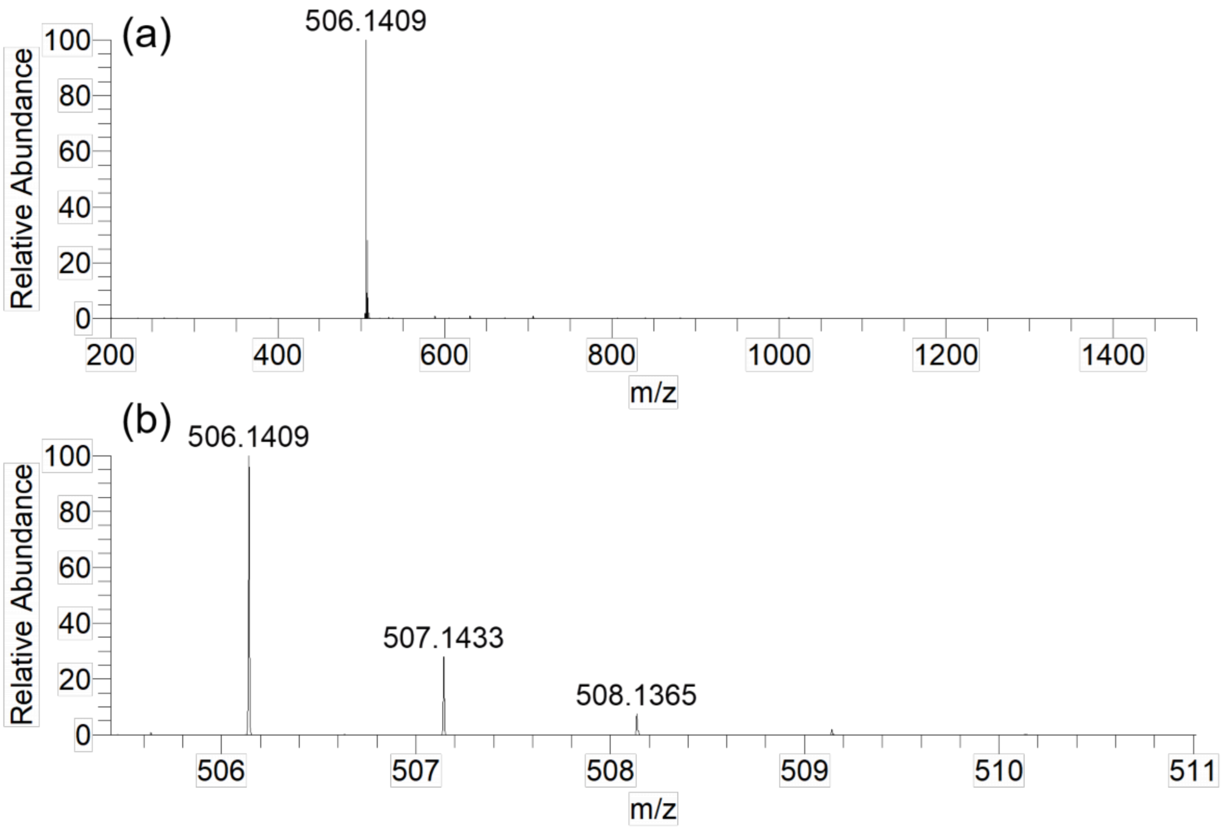

Figure S7. Mass spectrum of **1** extracted from the UHPLC-PDA-MS analysis shown in Figure S2. (a) Full mass spectrum: m/z observed: 506.1409 $[M+H]^+$. (b) Isotopic ion peaks of $[M+H]^+$. Monoisotopic m/z calculated: 506.1414. Monoisotopic m/z observed: 506.1409.

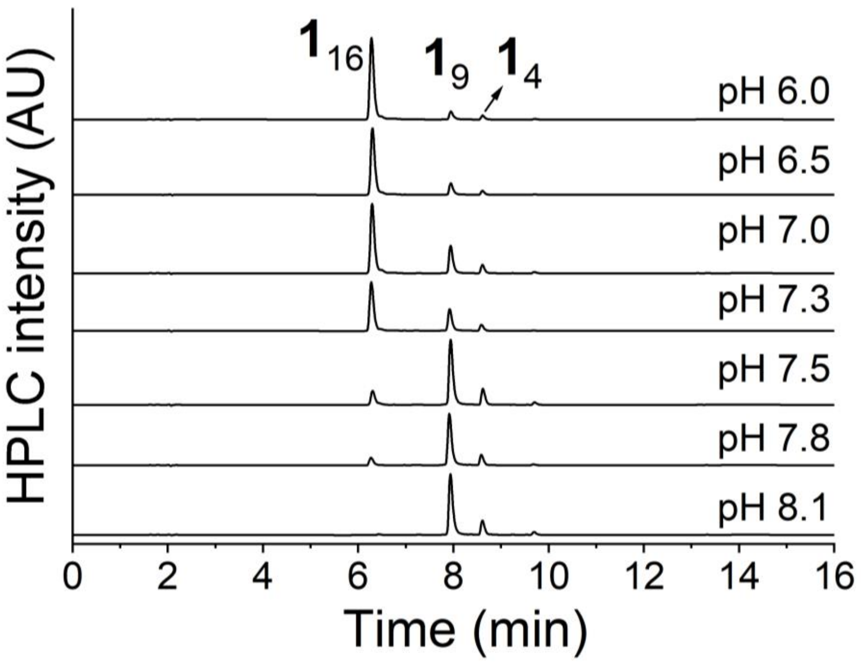

Figure S8. UHPLC chromatograms of libraries prepared from **1** in 25 mM phosphate buffer solution at different pHs.

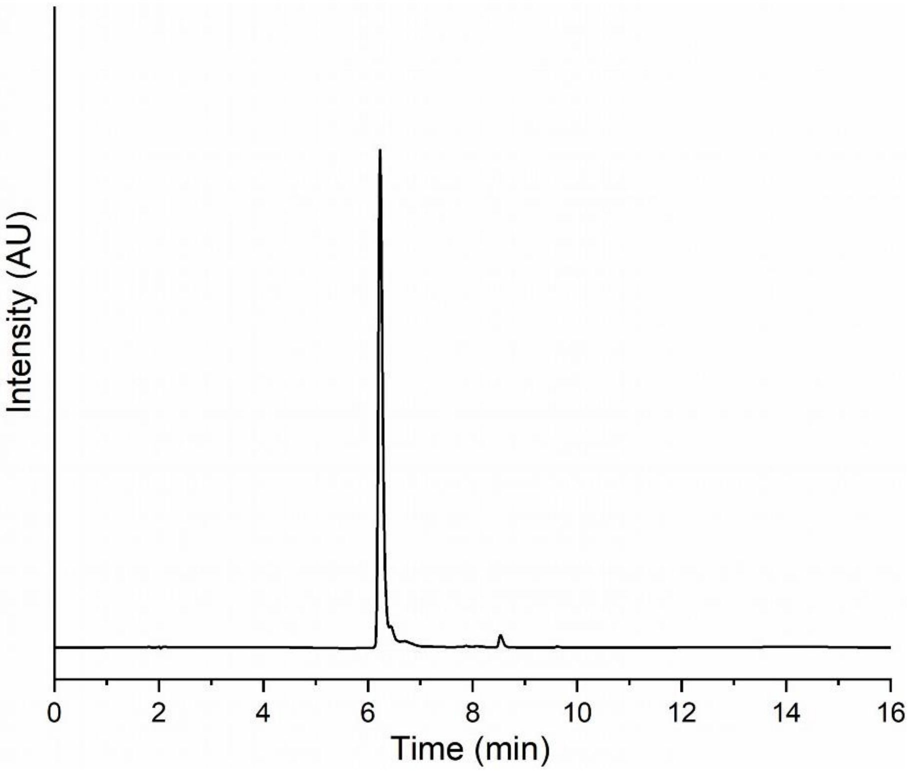

Figure S9. UHPLC analysis of purified $\mathbf{1}_{16}$.

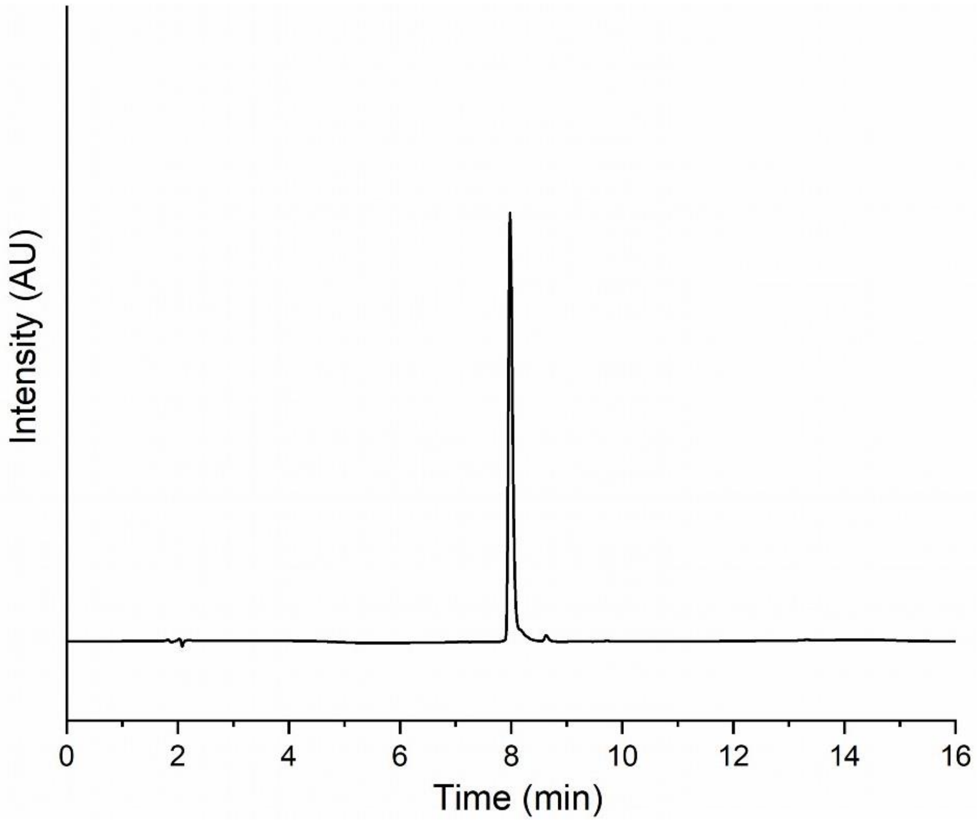

Figure S10. UHPLC analysis of purified $\mathbf{1}_9$.

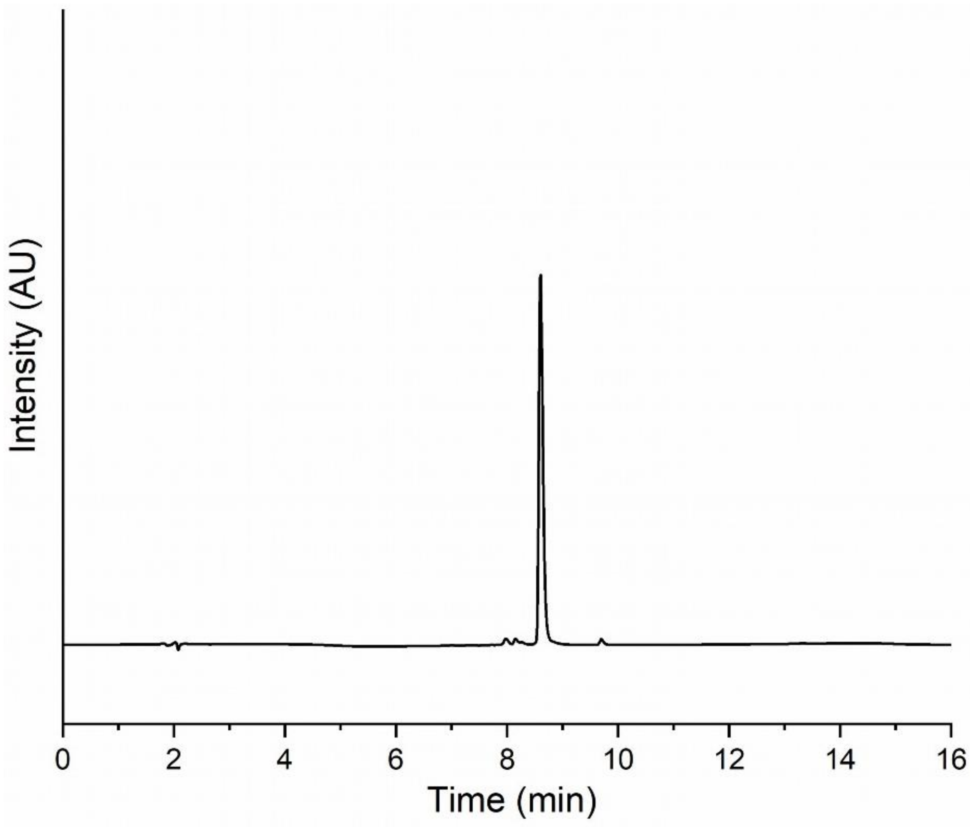

Figure S11. UHPLC analysis of purified $\mathbf{1}_4$.

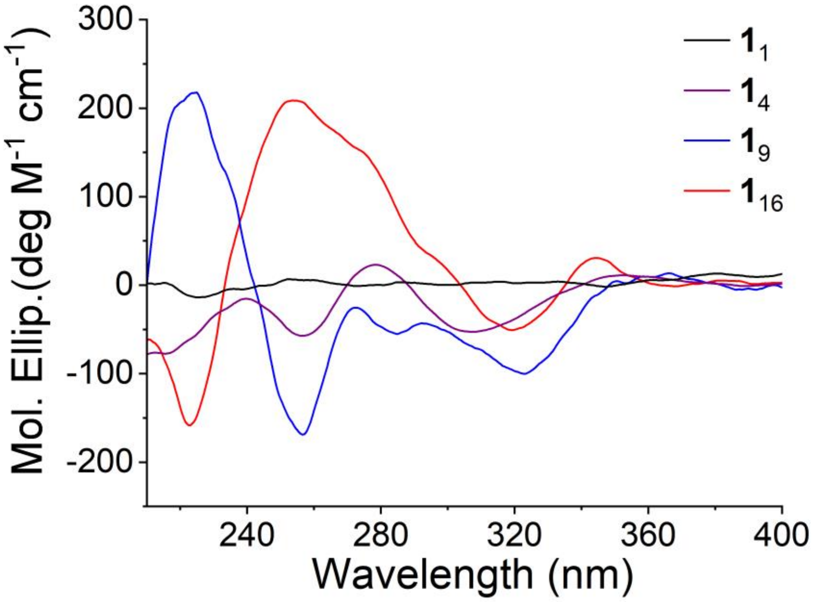

Figure S12. CD spectra of **1**, **1**$_4$, **1**$_9$, and **1**$_{16}$. The concentration of all samples was 0.10 mM in building block. Molar ellipticity is expressed in units of building block **1**.

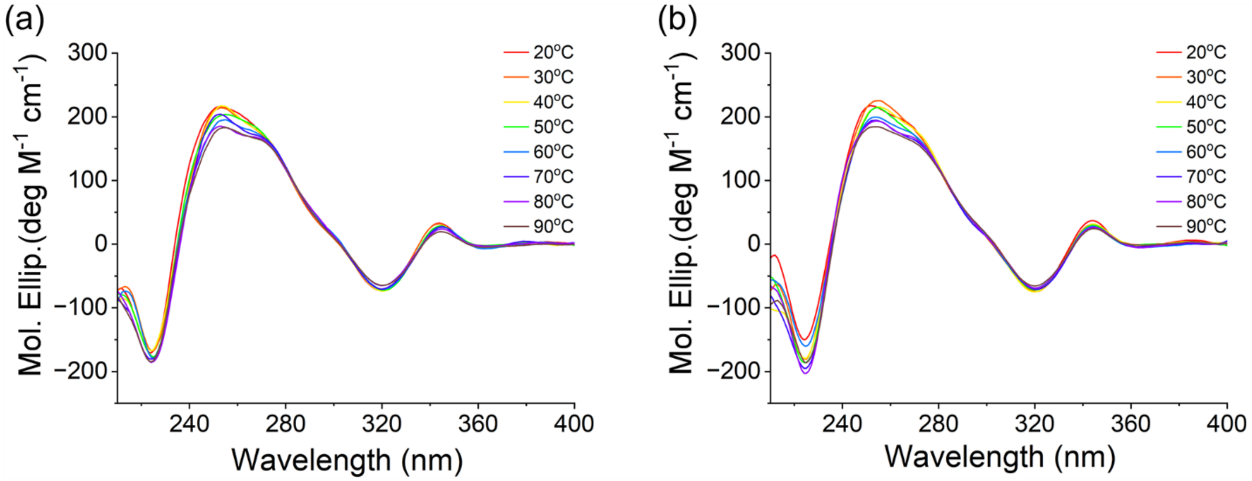

Figure S13. Variable temperature CD spectra of $\mathbf{1}_{16}$ (a) from 20 to 90 °C, and (b) subsequent 20-90 °C temperature increase of the same sample after cooled to 20 °C.

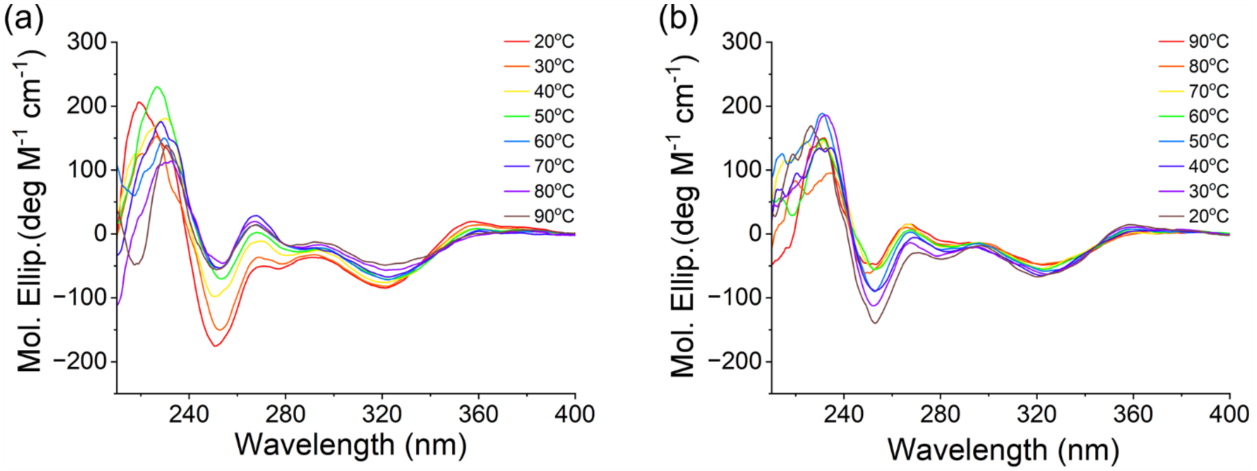

Figure S14. Variable temperature CD spectra of $\mathbf{1}_9$ (a) from 20 to 90 °C, and (b) from 90 to 20 °C.

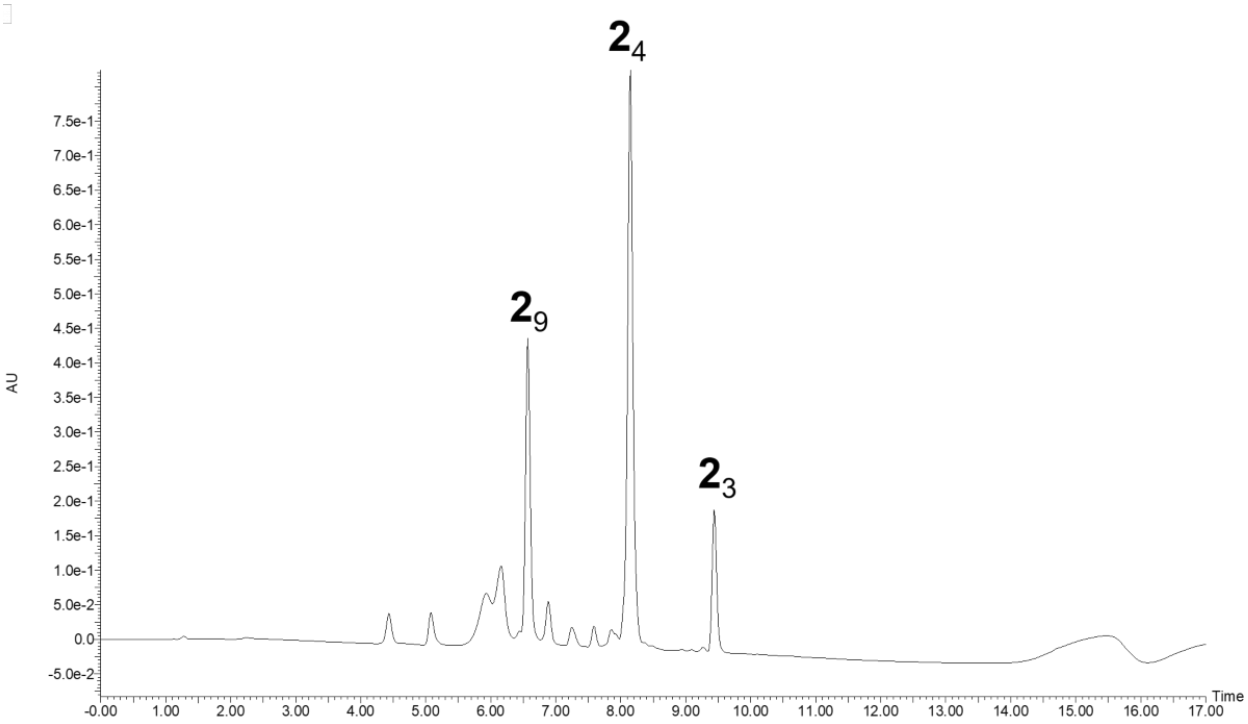

Figure S15. UPLC-PDA-MS analysis of the DCL made from 1.0 mM building block **2** in 25 mM phosphate buffer (pH 8.2) at day 2. Wavelength: 254 nm.

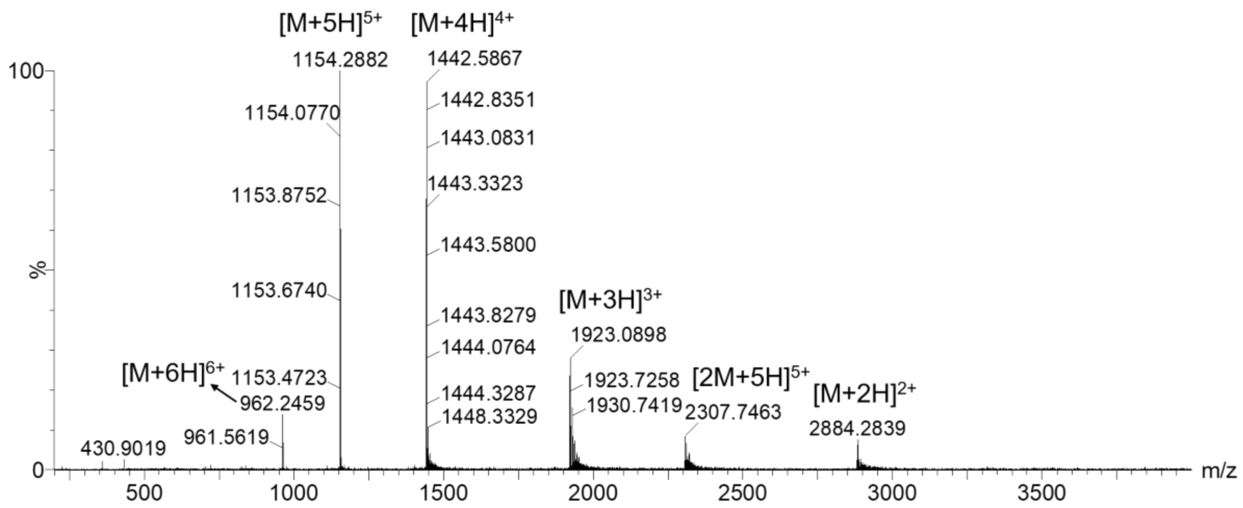

Figure S16. Mass spectrum of **2**$_9$ extracted from the UPLC-PDA-MS analysis shown in Figure S15. m/z calculated for **2**$_9$: 1442.41 $[M+4H]^{4+}$. m/z observed: 1442.59 $[M+4H]^{4+}$.

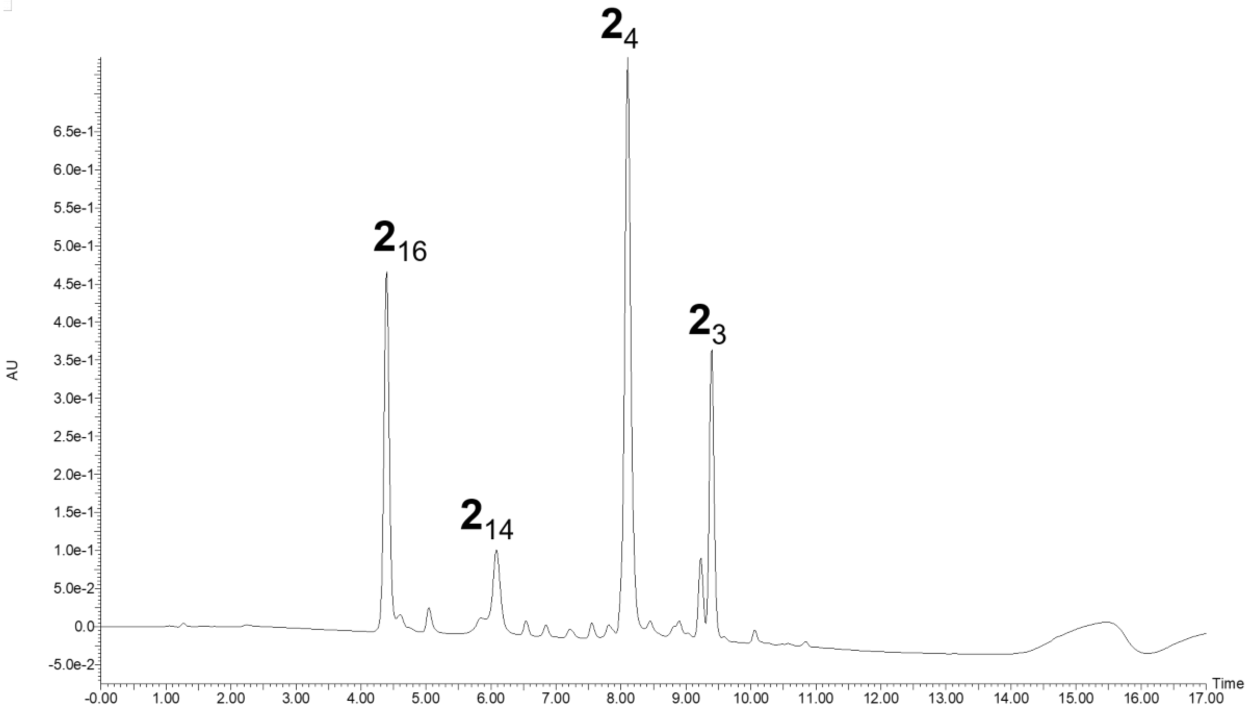

Figure S17. UPLC-PDA-MS analysis of the DCL made from 1.0 mM building block **2** in 25 mM phosphate buffer (pH 6.0) at day 2. Wavelength: 254 nm.

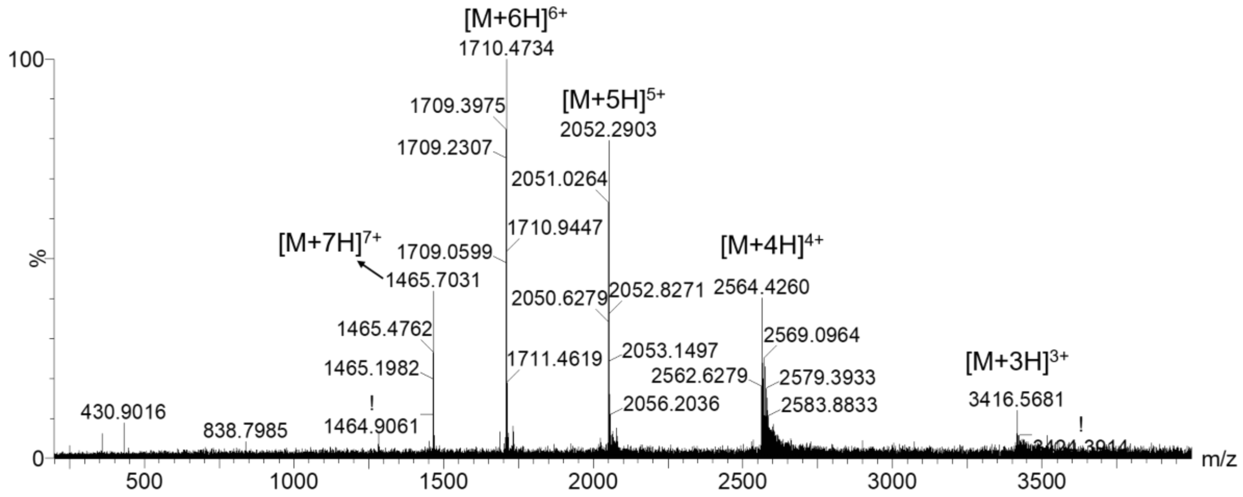

Figure S18. Mass spectrum of **2**$_{16}$ extracted from the UPLC-PDA-MS analysis shown in Figure S17. m/z calculated for **2**$_{16}$: 1709.48 $[M+6H]^{6+}$. m/z observed: 1709.40 $[M+6H]^{6+}$.

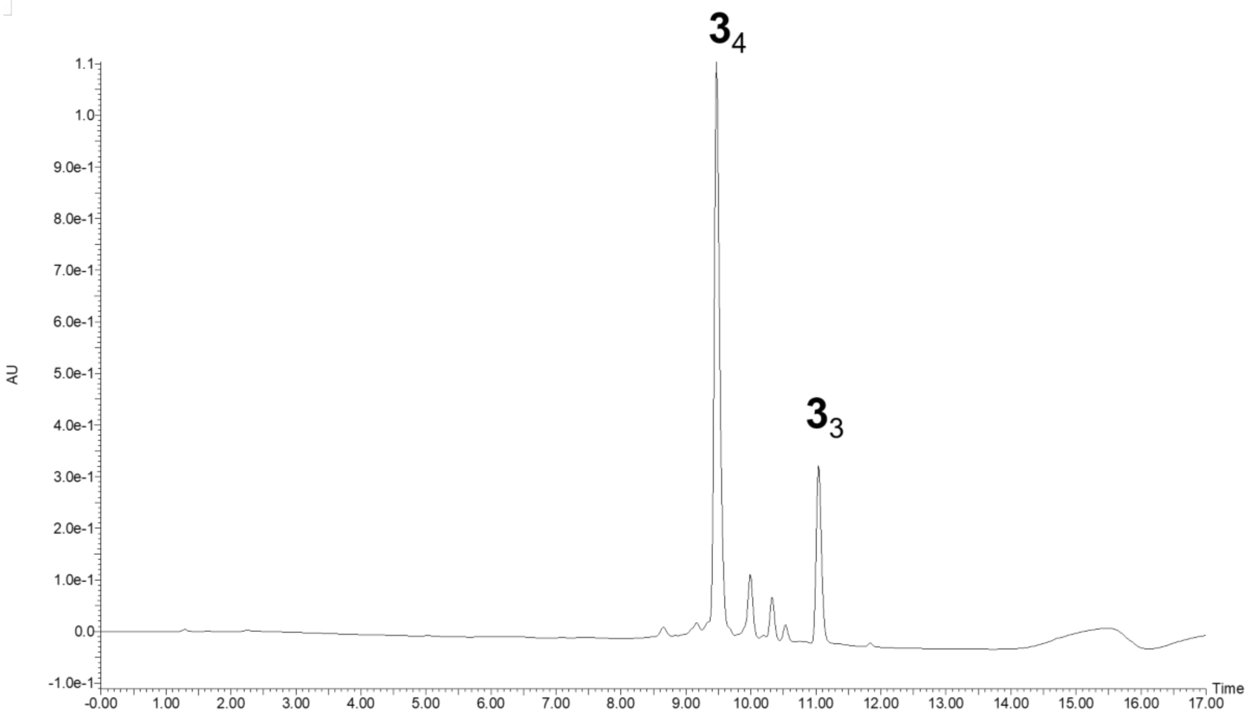

Figure S19. UPLC-PDA-MS analysis of the DCL made from 1.0 mM building block **3** in 25 mM phosphate buffer (pH 8.2) at day 2. Wavelength: 254 nm.

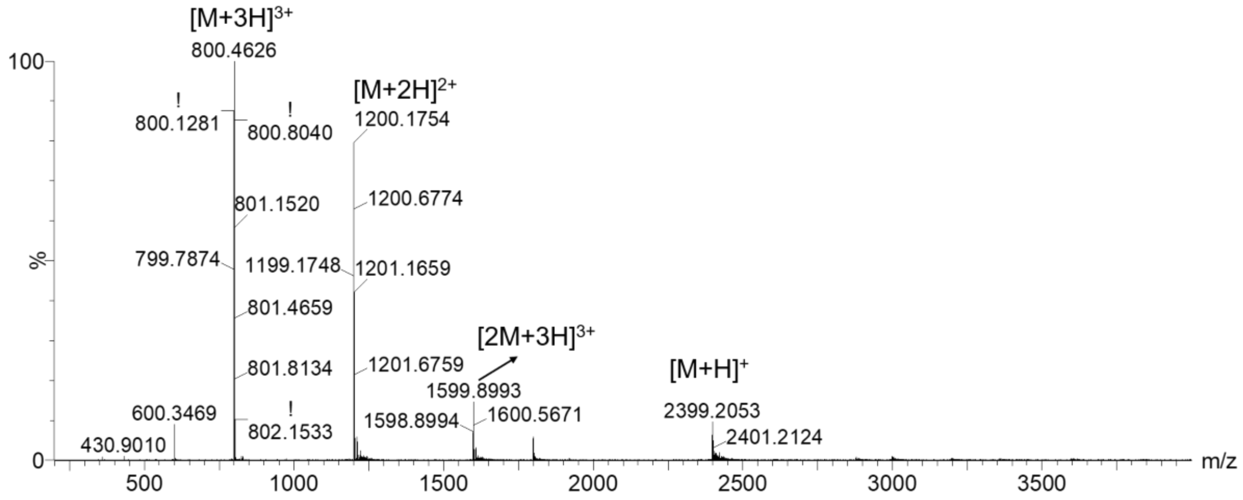

Figure S20. Mass spectrum of $\mathbf{3}_4$ extracted from UPLC-PDA-MS analysis shown in Figure S19. m/z calculated for $\mathbf{3}_4$: 800.16 [M+3H]$^{3+}$. m/z observed: 800.12 [M+3H]$^{3+}$.

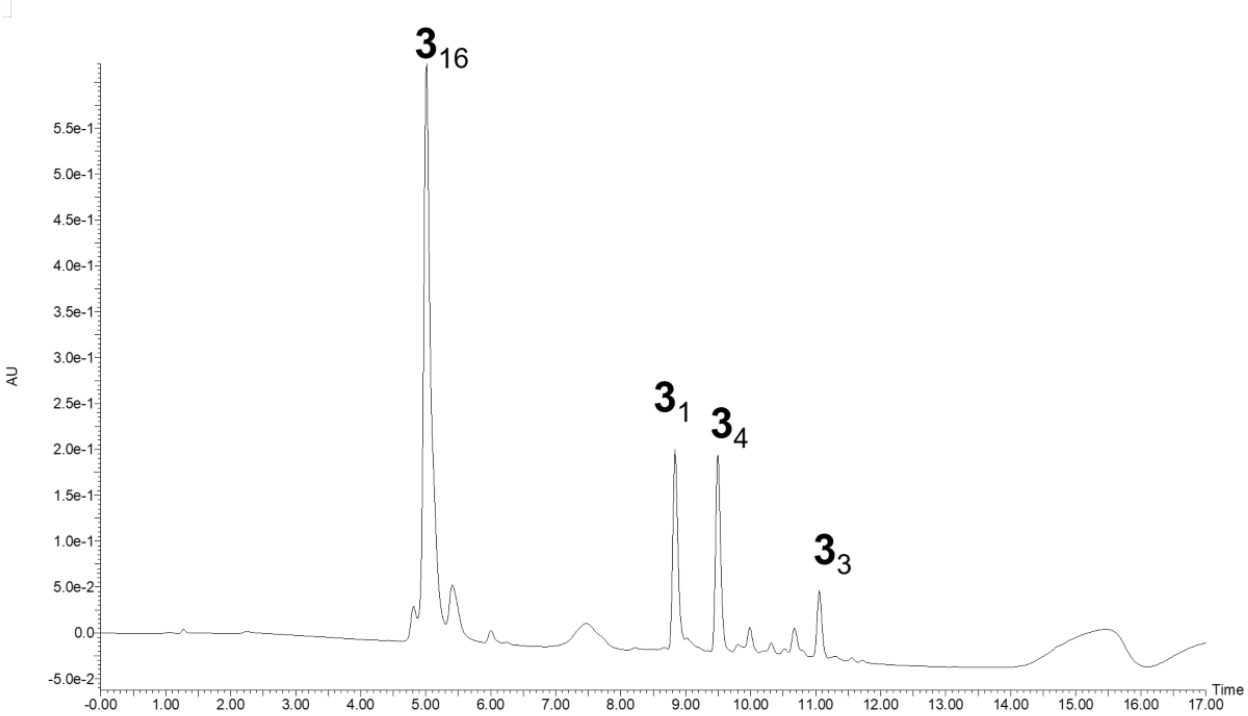

Figure S21. UPLC-PDA-MS analysis of the DCL made from 1.0 mM building block **3** in 25 mM phosphate buffer (pH 6.0) at day 2. Wavelength: 254 nm.

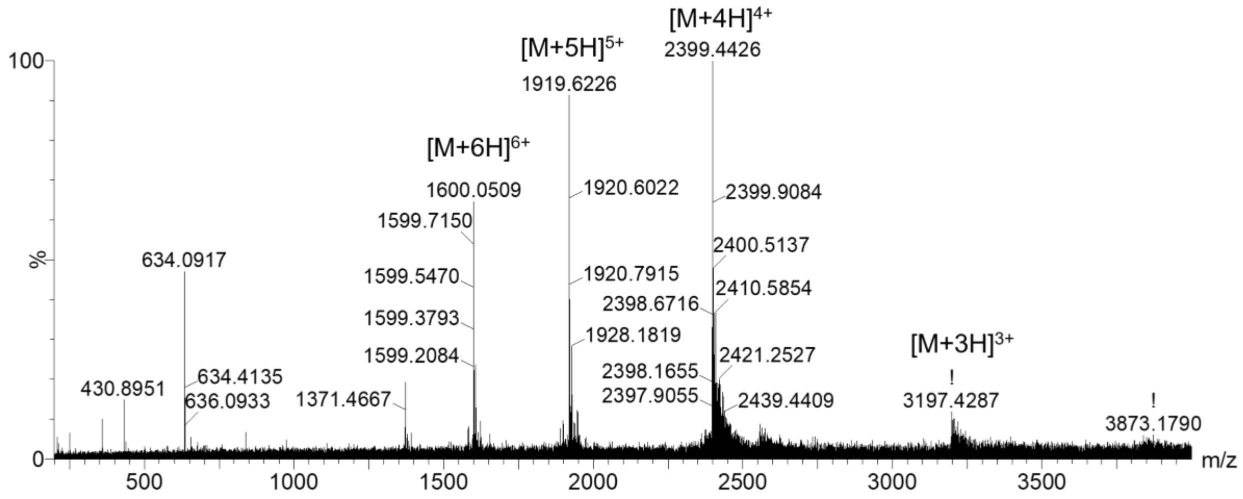

Figure S22. Mass spectrum of **3**$_{16}$ extracted from the UPLC-PDA-MS analysis shown in Figure S21. m/z calculated for **3**$_{16}$: 1919.57 $[M+5H]^{5+}$. m/z observed: 1919.62 $[M+5H]^{5+}$.

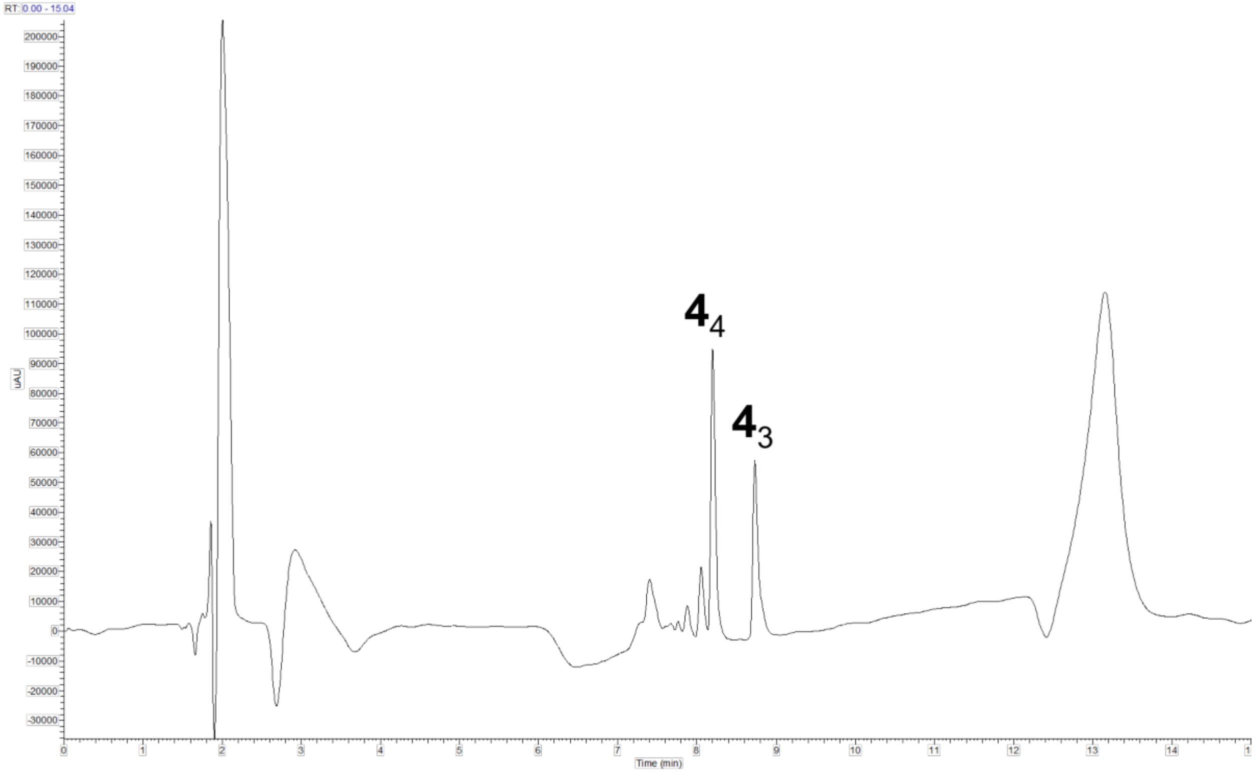

Figure S23. UHPLC-ion trap-MS analysis of the DCL made from 1.0 mM building block **4** in 25 mM phosphate buffer (pH 8.2) at day 2. Wavelength: 254 nm.

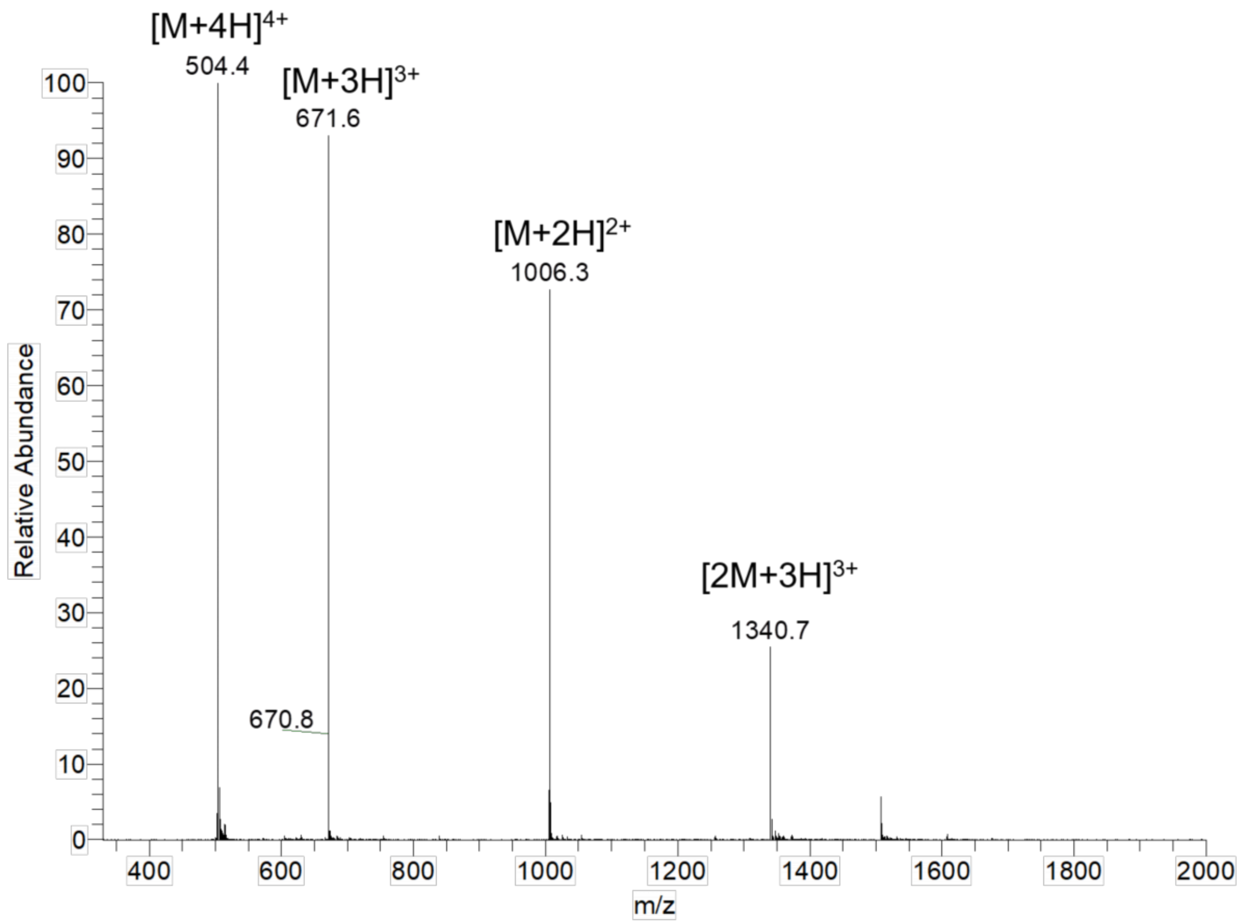

Figure S24. Mass spectrum of **4**$_4$ extracted from the UHPLC-PDA-MS analysis shown in Figure S23. m/z calculated for **4**$_4$: 671.1 $[M+3H]^{3+}$. m/z observed: 671.6 $[M+3H]^{3+}$.

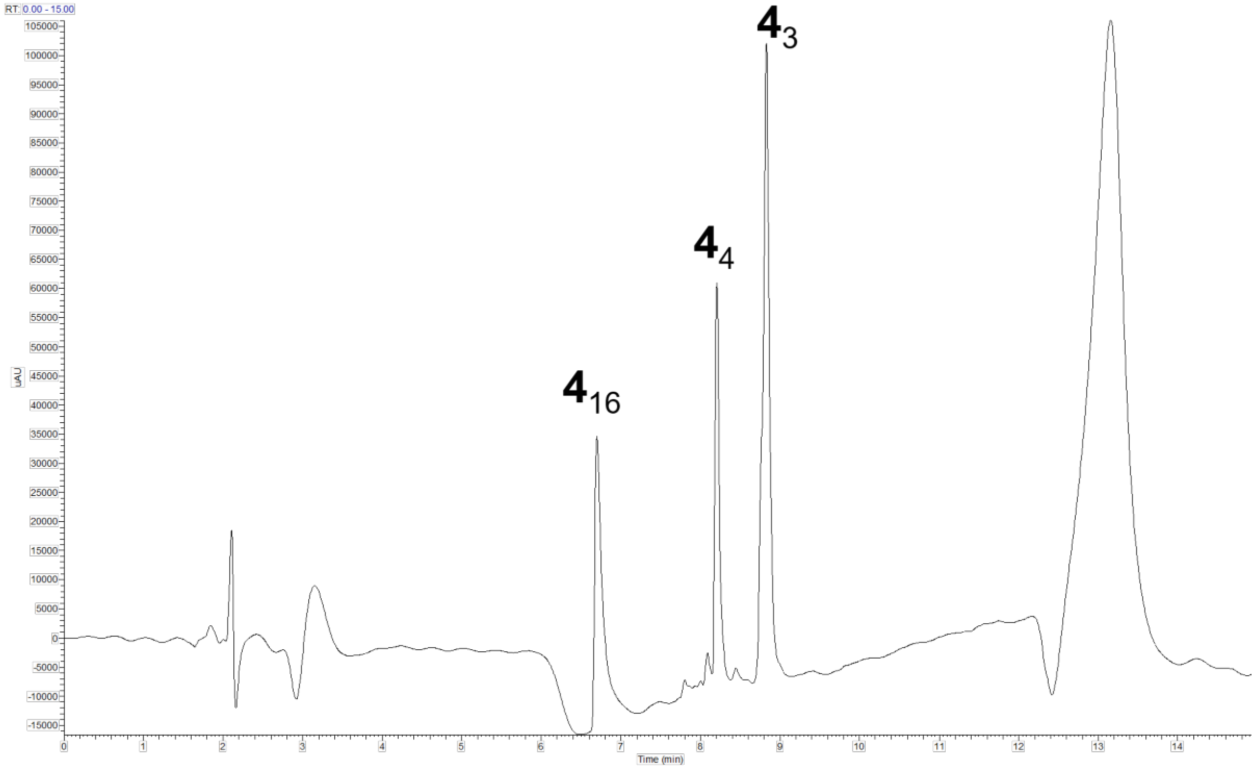

Figure S25. UHPLC-PDA-MS analysis of the DCL made from 1.0 mM building block **4** in 25 mM phosphate buffer (pH 6.0) at day 2. Wavelength: 254 nm.

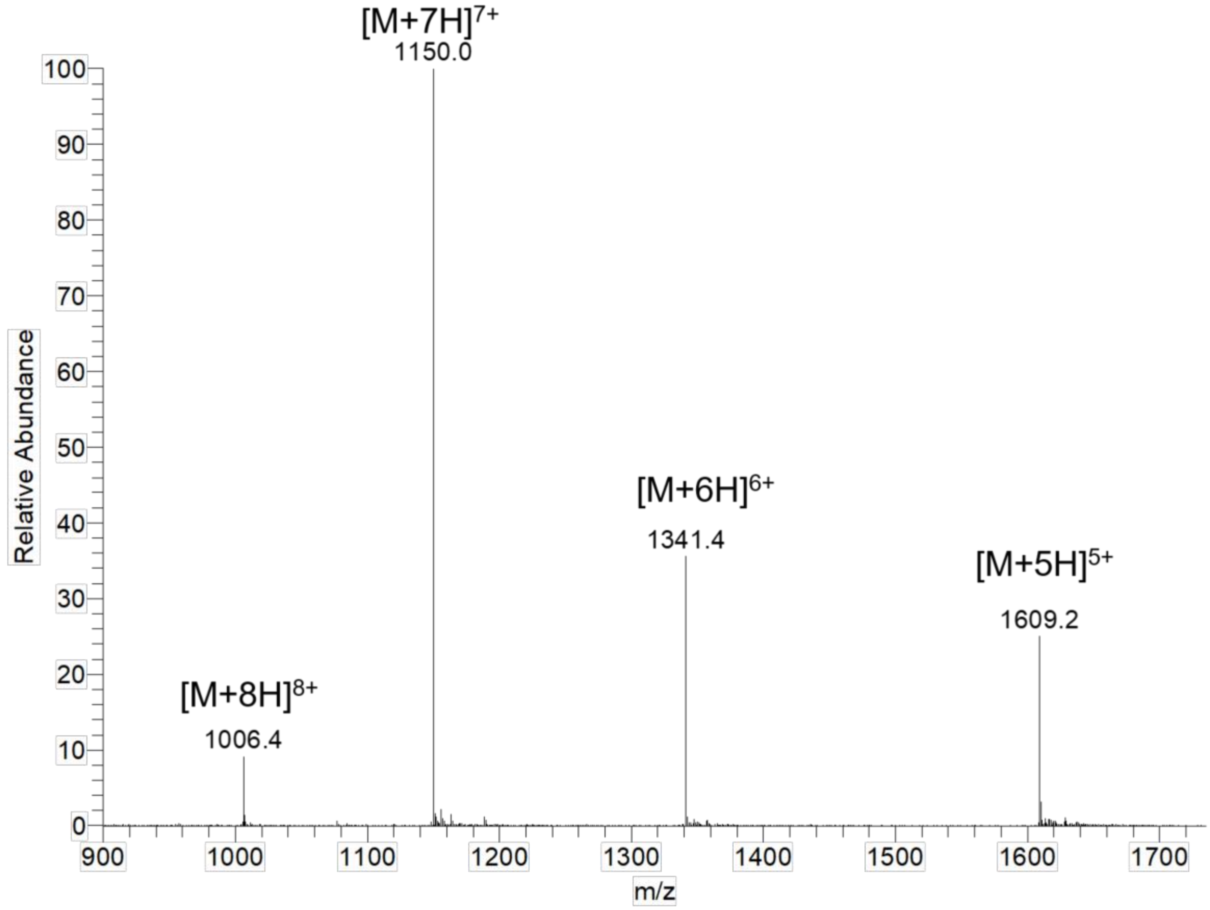

Figure S26. Mass spectrum of $\mathbf{4}_{16}$ extracted from the UHPLC-PDA-MS analysis shown in Figure S25. m/z calculated for $\mathbf{4}_{16}$: 1149.7 $[M+7H]^{7+}$. m/z observed: 1150.0 $[M+7H]^{7+}$.

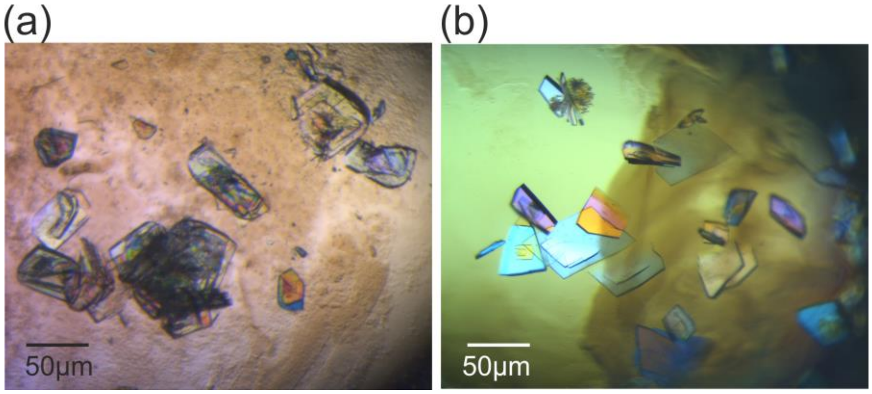

Figure S27. Crystals of (a) L/D-$\mathbf{1}_9$ and (b) L-$\mathbf{1}_{16}$ observed under crossed polarizing microscope.

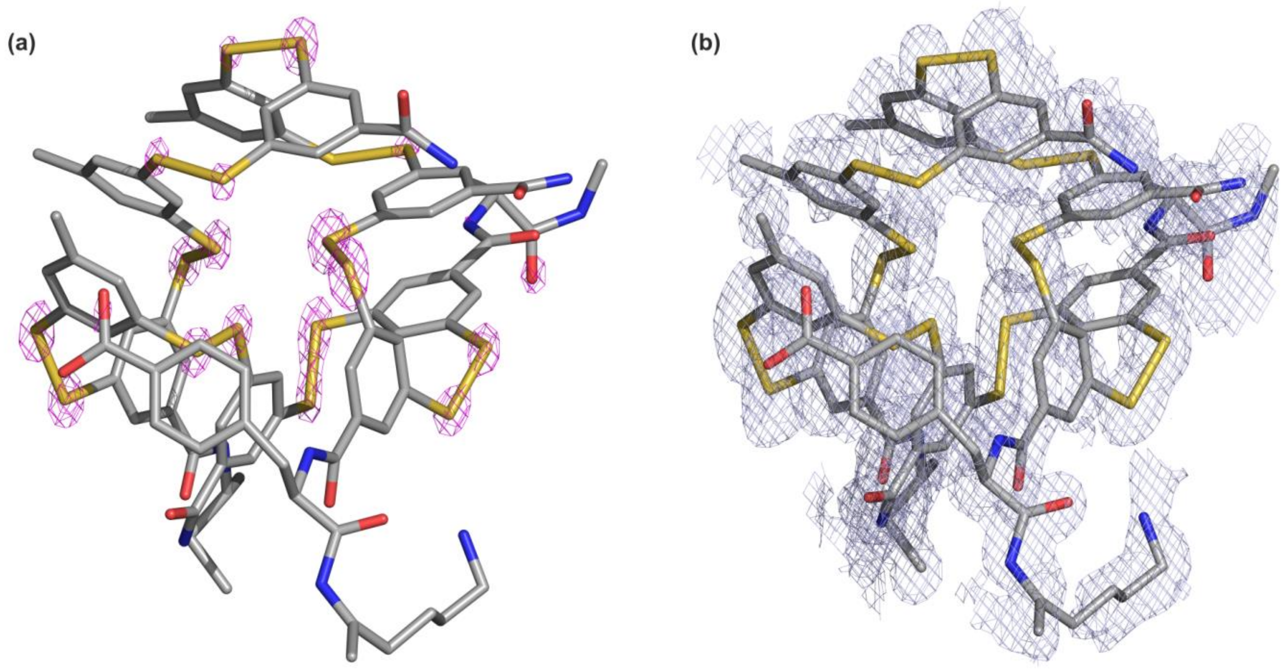

Figure S28. Sigma weighted $2F_o$-F_c electron density maps superimposed on L-**1**$_9$ macrocycle from the crystal structure of L/D-**1**$_9$. (a) Magenta mesh, contoured at 5 σ level shows the position of sulfur atoms and (b) grey mesh, contoured at 1 σ level shows the shape of macrocycle.

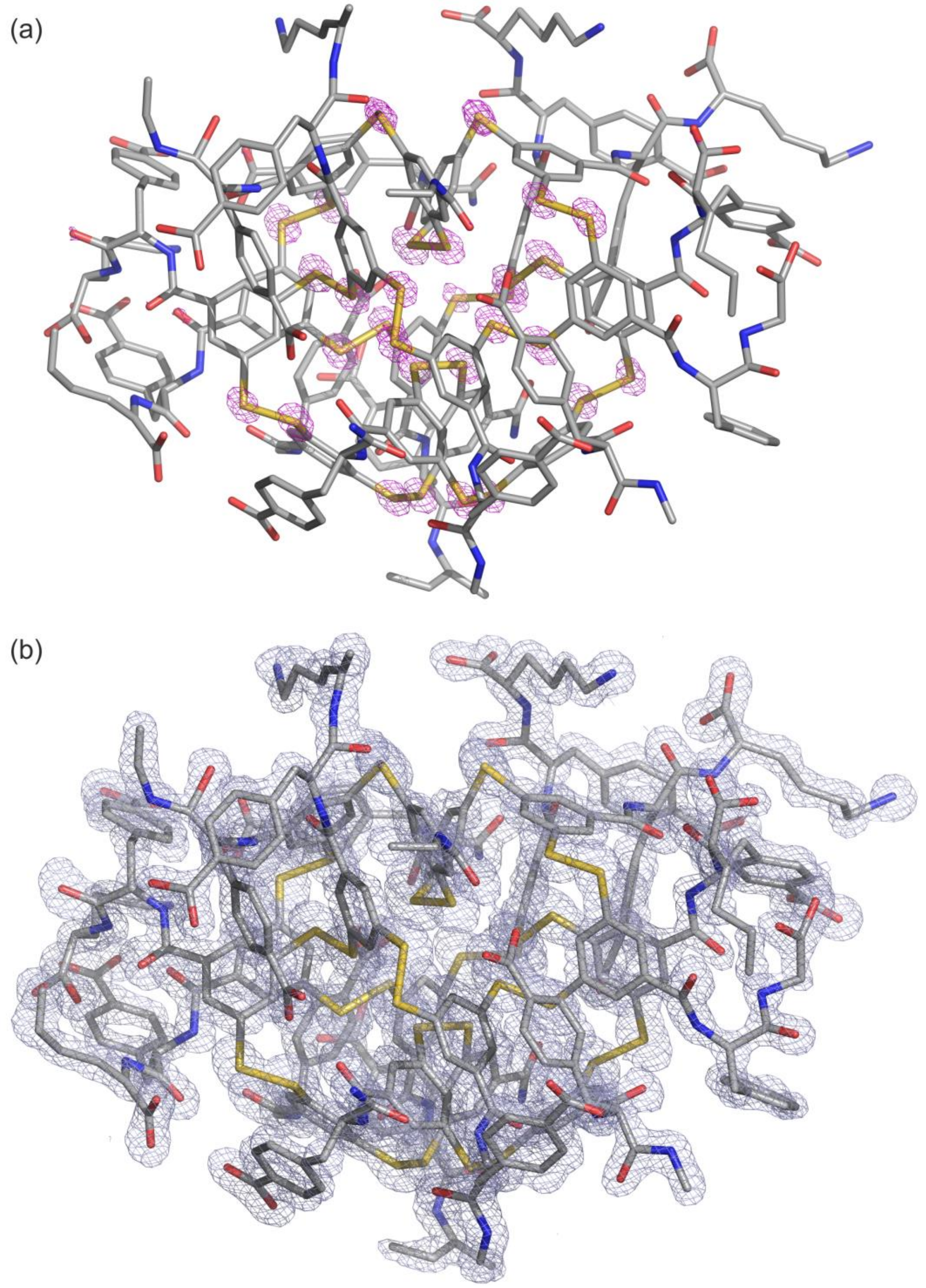

Figure S29. Sigma weighted $2F_o$-F_c electron density maps superimposed on L-**1**$_{16}$ macrocycle. (a) Magenta mesh, contoured at 6 σ level shows the position of sulfur atoms and (b) grey mesh, contoured at 1 σ level shows the shape of macrocycle.

3. References

(1) Otto, S.; Furlan, R. L. E.; Sanders, J. K. M., Selection and amplification of hosts from dynamic combinatorial libraries of macrocyclic disulfides. *Science* **2002,** *297*, 590-593.

(2) Dawson, S. J.; Hu, X.; Claerhout, S.; Huc, I., Solid phase synthesis of helically folded aromatic oligoamides. *Methods Enzymol.* **2016,** *580*, 279-301.

(3) Nurizzo, D.; Mairs, T.; Guijarro, M.; Rey, V.; Meyer, J.; Fajardo, P.; Chavanne, J.; Biasci, J. C.; McSweeney, S.; Mitchell, E., The ID23-1 structural biology beamline at the ESRF. *J. Synchrot. Radiat.* **2006,** *13*, 227-238.

(4) Evans, P., Scaling and assessment of data quality. *Acta Crystallogr. Sect. D-Struct. Biol.* **2006,** *62*, 72-82.

(5) Evans, P. R.; Murshudov, G. N., How good are my data and what is the resolution? *Acta Crystallogr. Sect. D-Biol. Crystallogr.* **2013,** *69*, 1204-1214.

(6) Kabsch, W., Xds. *Acta Crystallogr. Sect. D-Biol. Crystallogr.* **2010,** *66*, 125-132.

(7) Vonrhein, C.; Flensburg, C.; Keller, P.; Sharff, A.; Smart, O.; Paciorek, W.; Womack, T.; Bricogne, G., Data processing and analysis with the autoPROC toolbox. *Acta Crystallogr. Sect. D-Struct. Biol.* **2011,** *67*, 293-302.

(8) Winn, M. D.; Ballard, C. C.; Cowtan, K. D.; Dodson, E. J.; Emsley, P.; Evans, P. R.; Keegan, R. M.; Krissinel, E. B.; Leslie, A. G. W.; McCoy, A.; McNicholas, S. J.; Murshudov, G. N.; Pannu, N. S.; Potterton, E. A.; Powell, H. R.; Read, R. J.; Vagin, A.; Wilson, K. S., Overview of the CCP4 suite and current developments. *Acta Crystallogr. Sect. D-Struct. Biol.* **2011,** *67*, 235-242.

(9) Sheldrick, G. M., SHELXT - Integrated space-group and crystal-structure determination. *Acta Crystallogr. Sect. A* **2015,** *71*, 3-8.

(10) Sheldrick, G. M., Crystal structure refinement with SHELXL. *Acta Crystallogr. Sect. C-Struct. Chem.* **2015,** *71*, 3-8.

(11) Dolomanov, O. V.; Bourhis, L. J.; Gildea, R. J.; Howard, J. A. K.; Puschmann, H., OLEX2: a complete structure solution, refinement and analysis program. *J. Appl. Crystallogr.* **2009,** *42*, 339-341.

(12) Emsley, P.; Lohkamp, B.; Scott, W. G.; Cowtan, K., Features and development of Coot. *Acta Crystallogr. Sect. D-Biol. Crystallogr.* **2010,** *66*, 486-501.

(13) Spek, A. L., Structure validation in chemical crystallography. *Acta Crystallogr. Sect. D-Struct. Biol.* **2009,** *65*, 148-155.

(14) Cianci, M.; Bourenkov, G.; Pompidor, G.; Karpics, I.; Kallio, J.; Bento, I.; Roessle, M.; Cipriani, F.; Fiedler, S.; Schneider, T. R., P13, the EMBL macromolecular crystallography beamline at the low-emittance PETRA III ring for high- and low-energy phasing with variable beam focusing. *J. Synchrot. Radiat.* **2017,** *24*, 323-332.
(15) Agilent. *CrysAlisPRO*; Agilent Technologies Ltd, Yarnton, Oxfordshire, England 2014.
